# Loss of heterozygosity of CYP2D6 enhances the sensitivity of hepatocellular carcinomas to talazoparib

**DOI:** 10.1016/j.ebiom.2024.105368

**Published:** 2024-10-04

**Authors:** Xiaonan Zhang, Natallia Rameika, Lei Zhong, Verónica Rendo, Margus Veanes, Snehangshu Kundu, Sandro Nuciforo, Jordan Dupuis, Muhammad Al Azhar, Ioanna Tsiara, Pauline Seeburger, Shahed Al Nassralla, Viktor Ljungström, Richard Svensson, Ivaylo Stoimenov, Per Artursson, Markus H. Heim, Daniel Globisch, Tobias Sjöblom

**Affiliations:** a Department of Immunology, Genetics and Pathology, Science for Life Laboratory, Uppsala University, SE-751 85, Uppsala, Sweden; b Department of Biomedicine, University Hospital and University of Basel, CH-4031, Basel, Switzerland; c Clarunis University Center for Gastrointestinal and Liver Diseases, CH-4002, Basel, Switzerland; d Department of Chemistry-BMC, Science for Life Laboratory, Uppsala University, SE-751 23, Uppsala, Sweden; e Uppsala Drug Optimization and Pharmaceutical Profiling Facility (UDOPP), SciLifeLab Chemical Biology Consortium Sweden (CBCS), Department of Pharmacy, Uppsala University, 751 23, Uppsala, Sweden; f Department of Pharmacy, Uppsala University, Husargatan 3, 751 23, Uppsala, Sweden

**Keywords:** Loss of heterozygosity, Loss of function, Hepatocellular carcinomas, CYP2D6 and talazoparib

## Abstract

**Background:**

Loss of heterozygosity (LOH) diminishes genetic diversity within cancer genomes. A tumour arising in an individual heterozygous for a functional and a loss-of-function (LoF) allele of a gene occasionally retain only the LoF allele. This can result in deficiency of specific protein activities in cancer cells, creating unique differences between tumour cells and normal cells of the individual. Such differences may constitute vulnerabilities that can be exploited through allele-specific therapies.

**Methods:**

To discover frequently lost genes with prevalent LoF alleles, we mined the 1000 Genomes dataset for SNVs causing protein truncation through base substitution, indels or splice site disruptions, resulting in 60 LoF variants in 60 genes. From these, the variant rs3892097 in the liver enzyme *CYP2D6* was selected because it is located within a genomic region that frequently undergoes LOH in several tumor types including hepatocellular cancers. To evaluate the relationship between CYP2D6 activity and the toxicities of anticancer agents, we screened 525 compounds currently in clinical use or undergoing clinical trials using cell model systems with or without CYP2D6 activity.

**Findings:**

We identified 12 compounds, AZD-3463, CYC-116, etoposide, everolimus, GDC-0349, lenvatinib, MK-8776, PHA-680632, talazoparib, tyrphostin 9, VX-702, and WZ-3146, using an engineered HEK293T cell model. Of these, talazoparib and MK-8776 demonstrated consistently heightened cytotoxic effects against cells with compromised CYP2D6 activity in engineered hepatocellular cancer cell models. Moreover, talazoparib displayed *CYP2D6* genotype dependent effects on primary hepatocellular carcinoma organoids.

**Interpretation:**

Exploiting the loss of drug-metabolizing enzyme gene activity in tumor cells following loss of heterozygosity could present a promising therapeutic strategy for targeted cancer treatment.

**Funding:**

This work was funded by 10.13039/501100006313Barncancerfonden (T.S, PR2022-0099 and PR2020-0171, X.Z, TJ2021-0111), 10.13039/501100002794Cancerfonden (T.S, 211719Pj and D.G, 222449Pj), 10.13039/501100004359Vetenskapsrådet (T.S, 2020-02371 and D.G, 2020-04707), and the Erling Persson Foundation (T.S, 2020-0037 and T.S, 2023-0113).

**Research in context**

**Evidence before this study**

Loss of heterozygosity (LOH) is a common genetic event in the development of cancer. In certain tumor types, LOH can affect more than 20% of the genome, entailing loss of allelic variation in thousands of genes. This reduction of heterozygosity creates genetic differences between tumor and normal cells. In the past decade, the concept of collateral lethality has emerged, wherein the loss of gene activity due to deletions or LOH during tumor evolution is exploited to induce cancer-specific cell death.^1^, ^2^, ^3^ However, few if any target genes have been presented that can be targeted using existing approved cancer drugs and none of these approaches have therefore impacted cancer care.

**Added value of this study**

In this work, we devise a strategy to identify suitable genes for collateral lethality targeting based on prevalent loss-of-function alleles and LOH in cancer genomes, and identify a clinically used drug that exploits tumour specific loss of activity of one such target gene, *CYP2D6*. Compared to other work in the field, we demonstrate that collateral lethality can be exploited by approved drugs already in clinical use. Further, it opens for repurposing of existing drugs for use in hepatocellular carcinoma with unmet medical need, using gene panel sequencing for precision medicine.

**Implications of all the available evidence**

Our study identified 60 loss-of-function variants across 60 genes and highlights *CYP2D6* as a promising target for LOH therapy. This motivates future clinical studies where the use of CYP2D6 activity-dependent anti-cancer drugs is guided by constitutional and tumour *CYP2D6* genotype.

## Introduction

Development of targeted anti-cancer therapy has mainly focused on molecular differences between cancer and normal cells, seeking to identify drug targets enabling selective killing of tumor cells. Such therapies alternatively target gain of function in oncogenes, or loss of function in tumor suppressor genes or members of a particular repair or signalling pathway where biological function has been altered.^4^ The limited number of oncogenes that can be therapeutically targeted,^5^ and the practical limitations of reactivating tumor suppressor genes sustain the challenge of finding drug targets for anti-cancer therapy. Approved drugs encompass a range of targeted treatments, including monoclonal antibodies that enhance the immune recognition of cancer cells (e.g., rituximab and cetuximab for non-Hodgkin lymphoma and colorectal cancer, respectively), tyrosine kinase inhibitors that hinder cancer cell growth (e.g., Imatinib, a BCR-Abl inhibitor), and inhibitors targeting protein complexes (such as proteasome inhibitors and mTOR inhibitors).^6^^,^^7^ However, the efficiency of most targeted anti-cancer drugs is impaired by the emergence of resistance. Recently, cancer vulnerabilities emerging from genomic losses have been explored. Collateral lethality approaches have been demonstrated with *ENO2*^8^ and *ME3*^9^ as promising targets in glioblastoma and pancreatic cancer, as many tumors have complete loss of the corresponding genomic regions during tumor development. Similarly, inhibition of the methylosome member *PRMT5* reduces the growth of tumors that have defective methionine metabolism due to loss of *MTAP*.^10^ Alternative approaches include the search for haploinsufficiency targets, where copy number losses in cancer cells sensitize them to further gene suppression. Examples include *PSMC2*^11^ and *SF3B1*,^12^ involved in proteasome and spliceosome complexes, which are frequently found in a hemizygous state in colorectal, breast or bladder cancers.

Loss of heterozygosity causes loss of genetic variation in chromosomally unstable tumors. Such genomic losses may encompass chromosome arms or entire chromosomes, affecting on average 20% of the cancer genome.^13^ Further, an average of 100 LoF variants are present in the genome of a healthy individual.^14^ Thus, cancers arising in individuals who carry one functional and one non-functional allele of a gene may retain only the non-functional allele after LOH. This reduction in heterozygosity can make cancer cells susceptible to allele-specific therapies. By mining the human genome for common SNVs affecting the functional domains of enzymes, we previously identified the loss of *NAT2* as a therapeutic target for colorectal cancer, specifically in cases where the remaining *NAT2* allele following LOH has reduced enzymatic activity.^3^

**Fig. 1:**
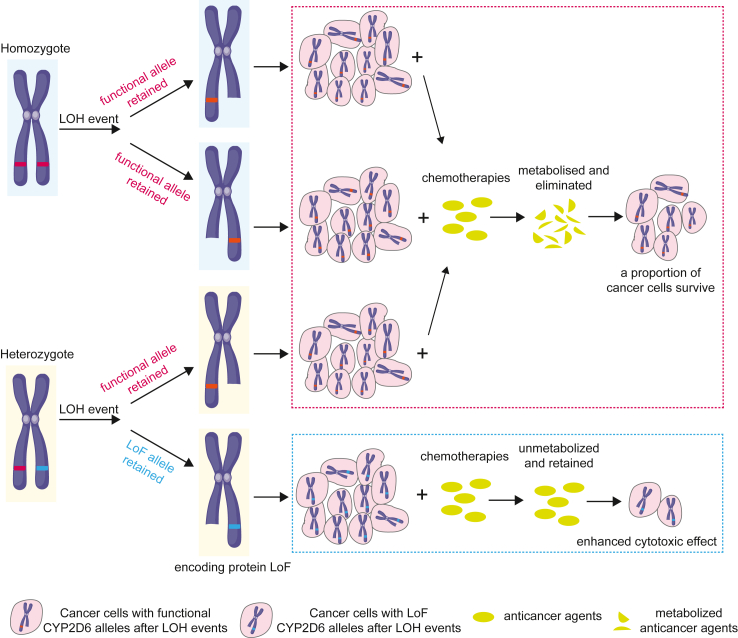
**A graphical summary illustrates the strategy used to identify CYP2D6 as a target for collateral lethality, driven by prevalent loss of heterozygosity (LOH) in cancer genomes**.

To find additional target genes for collateral lethality therapies, we here sought to identify genes with prevalent LoF alleles stemming from truncating, frameshifting or splice site mutations in data from the 1000 Genomes Project. We selected the rs3892097 variant in *CYP2D6* because it is located in a genomic region frequently affected by LOH in various tumor types, leading to the exclusive retention of the LoF allele in a subset of patient tumors following LOH. We established several different cell model systems and screened 525 anticancer agents, focusing on those currently in clinical use or undergoing clinical trials, to identify candidates with CYP2D6 activity dependent toxicity (Fig. 1).

## Methods

### Identification of prevalent loss-of-function variants

Data for human genetic variation were obtained from phase 1 samples of the 1000 Genomes project (Release 3, 2012-04-30). The consensus coding DNA sequence definitions of human protein coding exons were retrieved from the CCDS database (Release 15, 2013-11-29). The exon definitions were used to select all SNVs and indels in protein-coding regions and in splice junctions. The selection of splice site variants was restricted to mutations spanning the canonical dinucleotides belonging to splice donor or acceptor sites. Genetic variants with allele frequency <0.005 or >0.995 were discarded. The resulting set of loss-of-function variants was further filtered to enrich for exploitable targets in an LOH-based therapeutic approach. First, variants residing in olfactory, taste and opioid receptors, as well as in keratins and keratin-associated proteins were discarded (Supplementary Table S1). Second, only common variants having 10–90% heterozygosity in the population were retained. Third, all SNVs and indels causing truncation or frameshift and occurring in the first or last 10% of the protein-coding sequence were removed. The remaining set of variants was filtered according to expression profile, removing variants located in genes expressed at <300 AU (arbitrary units) in either normal or cancer tissues. Arbitrary units were normalized internally in the MediSapiens database, based on their method of scoring gene expression (IST Online [http://ist.medisapiens.com],). Fourth, SNVs and indels located in regions rarely undergoing loss of heterozygosity (<15% of cases) in common cancer types^13^ were discarded. To further validate the initial findings which were based on 1000 Genomes phase 1 data (Release 3, 2012-04-30), gnomAD was utilized as an additional resource for human genetic variation data (https://github.com/KalinNonchev/gnomAD_DB.git). Data was downloaded from the Broad Institute in the form of gnomAD v2.1.1, containing 261,942,336 total variants across 125,748 exomes and 15,708 whole genomes, all mapped to the GRCh37/hg19 reference sequence.^15^ Annotation of the data was achieved via the SnpEff software package. Impact annotations provided by SnpEff were used to initially reduce the volume of variants, retaining only variants annotated as having ‘high’ or ‘moderate’ impact.^16^ The consensus coding DNA sequence definitions of human protein coding exons were retrieved via NCBI RefSeq (Update, 2024-02-12) accessed through UCSCs genome browser.^17^ The exon definitions were used to select all SNVs and indels in protein-coding regions and in splice junctions. The selection of splice site variants was restricted to mutations spanning the canonical dinucleotides belonging to splice donor or acceptor sites. These variants were then filtered to remove those with allele frequency <0.5% and were additionally constrained to have heterozygosity between 10 and 90%. Variants outside splice sites occurring within the first or last 10% of the protein coding sequence were discarded alongside any which were not present in all gene transcripts.

Based on the available transcript definitions in UCSC, the SNVs and indels causing premature stop codons but not present in all known transcripts of the gene were removed (http://hgdownload.soe.ucsc.edu/goldenPath/hg19/database/knownGeneMrna.txt.gz]. The remaining variants were validated in the independent sets of data from HapMap Project (https://genome.ucsc.edu/cgi-bin/hgTrackUi?db=hg19&g=hapmapSnps], Exome Chip data (http://genome.sph.umich.edu/wiki/Exome_Chip_Design] and in our own whole-genome sequencing data from 32 individuals. Finally, variants for which no independent validation was available were removed from the selected list of putative targets.

### OMIM analysis

OMIM (Online Mendelian Inheritance in Man) analysis was performed on the initial set of 1398 LoF variants. The accession numbers for all available phenotypes are provided in Supplementary Table S2.

### Cell culture

HepG2 (#HB-8065) and HEK293T (#CRL-3216) cells were obtained from ATCC (Virginia, USA). HepG2 cells were maintained in EMEM medium (#30-2003, ATCC, USA) and HEK293T cells were maintained in DMEM medium (#41966029, Thermo Fisher Scientific, Massachusetts, USA) with the addition of 10% Foetal bovine serum (Thermo Fisher Scientific, Massachusetts, USA) and 1% penicillin-streptomycin (Thermo Fisher Scientific, Massachusetts, USA) at 37 °C in 5% CO_2_. All cell lines were authenticated by STR profiling (ATCC cell authentication service) and regularly checked for mycoplasma infection with MycoAlert mycoplasma detection kit (Lonza, Basel, Schweiz).

### Generation of cell models overexpressing wild-type or LoF CYP2D6

To generate stable clones overexpressing wild-type functional CYP2D6 (*CYP2D6∗1*) and LoF (*CYP2D6∗4*, rs3892097), lentiviral particles encoding FLAG-tagged wild-type CYP2D6∗1 and the variant CYP2D6∗4 (rs3892097) were cloned into the CS-A3525-Lv225-01 vector backbone (LabOmics, Belgium). The day before transduction, 50,000 cells were plated in a 24-well plate. Viruses were diluted in 250 μL of normal growth medium containing 7.5 μg/ml Sequa-Brene (Sigma, Missouri, USA) and added to each well. After 24 h of incubation, the virus-containing medium was replaced with fresh growth medium. After 48 h, transduced cells were selected with 1 μg/ml puromycin (Gibco, Montana, USA) for 10 days. For transient overexpression of electron donors POR and CYB5A, HEK293T cells stably overexpressing CYP2D6∗1 or CYP2D6∗4 were infected with adenovirus-POR (#SL100701, SignaGen, USA) and adenovirus-CYB5A (#SL100703, SignaGen, USA) for 3 d after cell plating in 24-well plates (Corning, Amsterdam, Netherlands) at a density of 600,000 cells/well in phenol red- and antibiotic-free DMEM medium with the indicated MOIs. Three wells were prepared for each condition. After 3 d of infection, cells from the same condition were collected and 20,000 cells/well were plated the day before determining CYP2D6 catalytic activity. The remaining cells were prepared for immunoblotting to confirm the expression level of POR and CYP5A after adenovirus infection. For stable overexpression of electron donors POR and CYB5A, HEK293T cells stably overexpressing either CYP2D6∗1 or CYP2D6∗4 were transduced with lentiviral particles encoding mCherry-POR (LPP-H3310-Lv166-050) and hygromycin-resistant CYB5A (LPP-Z0159-Lv115-050) following the procedure described above for generating stable CYP2D6 clones in HEK293T cells. Following lentiviral transduction of POR and CYB5A, cells were first selected for CYB5A in 150 μg/ml hygromycin for 10 days then the remaining cells were further selected by FACS using mCherry marker for POR. HEK293T parental cells were used as negative control during the FACS selection. Cells were collected after sorting and checked by immunoblotting after expansion. Generation of stable HepG2 cell clones overexpressing functional (CYP2D6∗1) and LoF CYP2D6 (CYP2D6∗4, rs3892097) followed the same procedure.

### Generation of CYP2D6 KO cells by CRISPR/Cas9 genome editing

HepG2 CYP2D6 KO cell pools were generated using CRISPR/Cas9 by Synthego (CA, USA) with the sgRNA sequence CGGCCCGAAACCCAGGAUCU. Approximately 500 single cells, one per well were prepared by FACS sorting in 96-well plates. After 3 weeks, colonies generated from every single cell were expanded in 24-well plates and further tested by qPCR using the forward primer 5′-TGGCGCGAGCAGAGGCGCTTC-3′ and reverse primer 5′-GTCCCCGTCCTCCTGCATATC-3’. CYP2D6 KO1 and KO2 were selected for subsequent experiments.

### RNA-sequencing and analysis

Cell pellets were collected and RNA was isolated, triplicates for each condition. All RNA samples passed sample quality control. RNA-SEQ was performed by Novogene using Illumina Sequencing PE150. Raw data in fastq format (raw reads) were first processed through an in-house perl script. In this step, clean data (clean reads) are obtained by removing adapter-containing reads, plot-N-containing reads, and low-quality reads from the raw data. Simultaneously calculate the Q20, Q30 and GC contents in the clean data. All downstream analyses are based on high-quality clean data. Further bioinformatics analysis procedures were performed according to the method instructions. For RNA-SEQ analysis, differential expression analysis of two conditions/groups (three biological replicates per condition) was performed using the DESeq2Rpackage (1.20.0). For GO enrichment analysis clusterProfiler (version 3.8.1) was used. RNA-SEQ raw data will be uploaded upon request.

### Western blot

Cells were collected and lysed in ice-cold RIPA lysis buffer and separated on 4–12% SDS-PAGE (#NP0336BOX, Thermo Fisher Scientific, USA). Proteins were transferred to nitrocellulose membranes and incubated at room temperature in 5% non-fat dry milk in 1 × PBST for 1 h. Membranes were then incubated overnight at 4 °C with anti-FlAG primary antibody (peptide sequence DYKDDDDK, #F3165, Mouse, Sigma) to detect the overexpressed CYP2D6∗1 (56 kDa) as well as CYP2D6∗4 (∼20 kDa) proteins, anti-CYP2D6 to detect endogenous CYP2D6 (#ab137426), anti-POR (#sc-25270, Mouse, Santa Cruz Biotechnology), anti-CYB5A (#NBP2-49284, Rabbit, NovusBio) and β-Actin (#sc-47778, Mouse, Santa Cruz Biotechnology). Next day, membranes were washed with 1 × PBST and incubated with anti-Rabbit (#31430, Thermo Fisher Scientific) or anti-Mouse secondary antibodies (#31460, Thermo Fisher Scientific) at a dilution of 1:5000 for 1 h. Immunoreactive bands were detected by FluorChem Fluorescent Western Imaging System from ProteinSimple (California, USA).

### Quantification of CYP2D6 catalytic activity

Cells were plated at a density of 20,000 cells per well in a 96-well plate. On the next day, incubations with the CYP2D6-specific substrate dextromethorphan (10 μM) were initiated both in the presence or absence of the CYP2D6 inhibitor quinidine (10 μM). Reactions were quenched by addition of 99.98% MeOH. The formation of dextrorphan after CYP2D6 metabolism was detected by LC-MS/MS on a XEVO TQ (Waters) instrument couples to an Acquity UPLC (Waters) using a HSS T3 column (1.7 μm, 2 × 50 mm). The mobile phases consisted of (A) 0.05% heptafluorobutyric acid and propionic acid, and (B) 0.05% heptafluorobutyric acid and propionic acid in acetonitrile.

Protein quantification was performed on cell pellets using Pierce BCA Protein Assay Kit (#23227, Thermo Scientific). The protein concentration for each pellet was calculated in order to adjust the volume of the solvents used for metabolite extraction. The extraction was performed using HPLC-grade CHCl_3_ and milli-Q water (4:4:2.85/sample solution: CHCl_3_:milli-Q water; fixed volume ratio). The sample tubes were shaken at 4 °C for 20 min in a Thermomixer (1400 rpm) and then centrifuged (5 min, 14,100 g). The aqueous phase was transferred to a new tube and dried under vacuum in a Speedvac. The remaining pellet was dissolved in water/acetonitrile (95/5, v/v) and the solution was transferred to LC vials for the UHPLC-MS/MS analysis.

The UHPLC-MS/MS analysis was performed in a Maxis II ETD Q-TOF mass spectrometer (Bruker Daltonics, Germany) using an electrospray ionization (ESI) source with an elute UHPLC (Bruker Daltonics, Germany) system. Milli-Q water with 0.1% formic acid was used as mobile phase A and LC-MS grade methanol with 0.1% formic acid was used as mobile phase B. The column temperature was kept at 40 °C, and the autosampler temperature was kept at 4 °C. The flow rate was set to 0.22 ml/min. The gradient used was as follows: 0–2 min, 0% B; 2–15 min, 0–100% B; 15–16 min, 100% B; 16–17 min, 100–0% B; 17–23 min, 0% B. The system was controlled using the Compass HyStar software package from Bruker (Bruker Daltonics, Germany). High-resolution mass spectra were acquired in positive mode at a mass range of *m*/*z* 50–1200. Data acquisition was performed in AutoMSMS mode (data dependent acquisition, DDA) with ramped collision energy from 20 eV to 50 eV. A solution of sodium formate [10 mM in a mixture of 2-propanol/water (1/1, v/v)] was used for internal calibration at the beginning of each run, in a segment between 0.10 and 0.31 min.

### Cell viability assay

Cell viability was determined either using CellTiter-Glo® luminescent cell viability assay medium (#G9681, Promega, Madison, USA) for 384-well plate format in screening described below or resazurin-based assay solution (Sigma–Aldrich) for drug candidates confirmation in 96-well plate format. To perform the resazurin-based cell viability assay, cells were incubated with compounds at indicated concentrations for 72 h at 37 °C then medium was removed from each well and replaced with resazurin solution for 2.5 h at 37 °C. The fluorescence intensity was read using the Victor 1420 multilabel counter (Wallac) at 560 nm excitation/590 nm emission. Percentage of cell viability was calculated as (fluorescence intensity of treated cells – fluorescence intensity of background)/(fluorescence intensity of DMSO treated control—fluorescence intensity of background) x 100%. To calculate the half-maximal inhibitory concentration (IC_50_), at least 5 different concentrations were made. The half-maximal inhibitory concentration (IC_50_) was calculated using dose–response inhibition (inhibition vs normalized response) analysis in GraphPad.

### Drug screening and hit confirmation in HEK293T cells

Two compound libraries, in total 525 drugs, were used for drug screening: (1) 147 FDA approved oncology drugs set (compound list) provided by the developmental therapeutics program at the National Cancer Institute (NCI/DTP); and (2) 378 kinase inhibitors set (compound list) provided by Chemical Biology Consortium Sweden (CBCS). Compounds (10 mM in DMSO) were spotted in assay ready plates (NUNC TC black optical bottom 384-well plates) with an Echo dispenser to a final volume of 40 nl per well, and 40 nl of DMSO was spotted in control wells. Compounds were tested at 10 concentrations: 0, 40, 80, 160, 315, 625, 1250, 2500, 5000 and 10,000 nM spotted in rows. HEK293T CYP2D6∗^1+POR+B5A^ and CYP2D6∗^4+POR+B5A^ cells were seeded in 60 μl DMEM medium supplemented with 10% FBS and 1% Pen-Strep, at a density of 3000 cells per well in assay ready plates and incubated at 37 °C, 5% CO_2_. After 72 h, 60 μl CellTiter-Glo® Luminescent Cell Viability Assay medium (#G9681, Promega, Madison, USA) was added to each well using a BioMek Nx robot. Cells were incubated with CellTiter-Glo for 30 min at 37 °C, 5% CO_2_ after which luminescent signal was measured. To normalize data, % viability was calculated for each datapoint as luminescent units (RLU) for compound/RLU DMSO control in respective compound. We then followed a similar criterion for hit compound selection,^18^ first excluding compounds with <20% decreased viability over DMSO control at 10 μM, then selecting compounds where % viability HEK293T CYP2D6∗^1+POR+B5A^/CYP2D6∗^4+POR+B5A^ at any concentration >1.2. To confirm the selected compounds, the HEK293T cell viability assay was repeated 2–3 times on with 5 concentrations representing the optimal dose response range. We retained compound for which % viability HEK293T CYP2D6∗^1+POR+B5A^/CYP2D6∗^4+POR+B5A^ >1.2. and IC_50_ HEK293T CYP2D6∗^1+POR+B5A^/CYP2D6∗^4+POR+B5A^ ≥ 2. After this step, 12 compounds were selected as hit candidates.

### Hit confirmation in HepG2 cell models

The selected 12 compounds were further tested on HepG2 cell models. Firstly, HepG2 parental cells were treated at 10 and 50 μM to determine the appropriate dose response range. Next, parental and CYP2D6 KO clones were seeded at a density of ∼10,000 cells per well in 96 well plates, treated with indicated concentrations of compounds and incubated at 37 °C, 5% CO_2_ for 72 h. The confirmed compounds were further tested using HepG2 cells either overexpressing *CYP2D6∗1* or *CYP2D6∗4*.

### CYP activity assays

The activity of different CYPs was detected following the protocol of P450-Glo™ Assay Kits of Promega (#V8421, V8791, V9001). Briefly, HepG2 cells (#HB-8065) obtained from ATCC and HepG2-CYP cells (HepG2-CYP1A2, HepG2-CYP2C9, HepG2-CYP3A4) obtained from Hera Biolabs (KY, USA) were seeded in 96-well plates at a density of 12,000 cells/well and cultured for 72 h. The culture medium was then replaced with fresh medium or buffer containing corresponding luminogenic CYP substrates, including luciferin-1A2 for CYP1A2, luciferin-H for CYP2C9, and luciferin-IPA for CYP3A4. CYP1A2 inhibitor α-naphthoflavone (5 μM), CYP2C9 inhibitor sulfaphenazole (5 μM) and CYP3A4 inhibitor ketoconazole (2.5 μM) were also added to the HepG2-CYP cells with the luminogenic CYP substrates. After incubation for an appropriate time as recommended in the instructions, equivalent culture medium/buffer and luciferin detection reagent (25 μL each) were transferred to an opaque 96-well white luminometer plate and incubated at room temperature for 20 min. Then luminescence was detected using a CLARIOstar Reader (BMG LABTECH, Germany).

### Generation of spheroids

Spheroids were prepared using a modification of our previously described method.^19^ 10,000 cells/well were plated in 96 well plates. Plates were incubated for 4 day before drug exposure. Spheroids were then treated with indicated concentrations of compounds for 72 h followed by image acquisition and resazurin assay. The volumes of spheroids were calculated as V = 4/3 × πr^3^.

### HCC organoid generation and expansion

HCC tumor tissues for organoid generation were collected from patients undergoing diagnostic liver biopsy or surgical resection at the University Hospital Basel. Written informed consent was obtained from all patients. The study was approved by the local ethics committee (protocol numbers EKNZ 2014-099 and BASEC 2019-02118). HCCOs were generated as described previously.^20^ Briefly, tumor tissue was dissociated to small cell clusters and seeded into basement membrane extract type 2 (BME2, R&Dsystems, Cat. No. 3533-005-02). After BME2 polymerization, expansion medium (EM) was added. The EM composition was Advanced DMEM/F-12 (Gibco, Cat. No. 12634010) supplemented with B-27 (Gibco, Cat. No. 17504001), N-2 (Gibco, Cat. No. 17502001), 10 mM Nicotinamide (Sigma, Cat. No. N0636), 1.25 mM N-Acetyl-l-cysteine (Sigma, Cat. No. A9165), 10 nM [Leu15]-Gastrin (Sigma, Cat. No. G9145), 10 μM Forskolin (Tocris, Cat. No. 1099), 5 μM A83-01 (Tocris, Cat. No. 2939), 50 ng/ml EGF (Peprotech, Cat. No. AF-100-15), 100 ng/ml FGF10 (Peprotech, Cat. No. 100-26), 25 ng/ml HGF (Peprotech, Cat. No. 100-39), 10% RSpo1-conditioned medium (v/v, homemade).

### Variant calling and phenotype prediction of human HCCOs

Variant detection was performed using two different variant callers, FreeBayes and BCFtools, with HumanG1Kv37 as reference genome. When calling variants using FreeBayes, minimum base quality was set to 20 and minimum mapping quality was set to 1. No filter was used for variant detection with BCF tools. Considering that no formaldehyde was applied to the cells, i.e., the risk for preparation-induced DNA-damage is low, exclusion of variants with strand bias >90% was used as a filtering step. Additionally, quality score <20 was excluded. Subsequently, we searched for known *CYP2D6* variants in our data set. Known *CYP2D6* genotypes, their associated alleles and their effect on CYP2D6 function were obtained from Human Cytochrome P450 (CYP) Allele Nomenclature Committee. Certain genotypes are specific for certain haplotypes, meaning that their presence defines it. However, there are many reported *CYP2D6* genotypes that occur in several alleles, which, together with the fact that short read sequencing was used, complicates the process of predicting the variant alleles. To circumvent this issue, the combination of variants detected in each sample and to some extent the variant allele frequency was used to assess the probability of the detected variants being present on the same allele, and therefore predicting the *CYP2D6* star alleles. After determining the likely diplotype, the phenotype was predicted according to the definition^21^: UM (ultrarapid metabolizers) if they carried >2 fully functional alleles (∗*1*, ∗*2*, ∗*33*, ∗*35, ∗39, ∗53*), EM (extensive, or normal, metabolizers) if they possessed 2 fully functional alleles or 1 fully functional and a semi-active allele (∗*9*, ∗*10*, ∗*17*, ∗*29*, ∗*36*, ∗*41, ∗53, ∗54, ∗59, ∗72, ∗84*), IM (intermediate metabolizers) if they had only 1 fully functional allele and a null allele (*∗3–∗8, ∗11, ∗12, ∗14–∗16, ∗18–∗21, ∗38, ∗40–∗42, ∗44, ∗51, ∗56, ∗57, ∗60, ∗62, ∗68, ∗69, ∗92, ∗96, ∗99–∗101, ∗114*) or 2 semi-functional alleles, PM (poor metabolizers) were determined to have 2 null alleles. Among the SNPs detected by both variant callers, two different allele specific *CYP2D6* variants were found. These included rs28371717 which is unique for *CYP2D6∗33* and does not alter the function of *CYP2D6*, as well as rs3892097 which is unique for *CYP2D6∗4* and results in decreased function of *CYP2D6* (Supplementary Table S3).

### Drug treatment of human HCCOs

For HCCO drug sensitivity assays 11 different HCCO lines were used. HCCOs were dissociated into single cells using 0.25% Trypsin–EDTA (Gibco, Cat. No. 25200056) and plated at 1000 cells/well in 384-well plates. After 3 days, drugs were added using a D300e digital dispenser (Tecan) and the HCCOs treated for a total of 5 days. Cell viability was measured using the CellTiter-Glo 3D reagent (Promega, Cat. No. G9681) according to the manufacturer's instructions. Results were normalized to vehicle (DMSO) and curve fitting was performed using GraphPad software and the nonlinear regression fitting (four parameters model).

### Ethics declarations

HCC tumor tissues for organoid generation were collected from patients undergoing diagnostic liver biopsy or surgical resection at the University Hospital Basel. Written informed consent was obtained from all patients. The study was approved by the local ethics committee (protocol numbers EKNZ 2014-099 and BASEC 2019-02118).

### Statistical analysis

Statistical parameters and tests are reported in the Figures and corresponding Figure Legends. Statistical analysis was done using GraphPad Prism (GraphPad Software Inc). Results were considered statistically significant when the p-value was less than 0.05 (p < 0.05).

### Data availability

The HepG2 parental and CYP2D6 KO RNA-SEQ raw data included in this study have been deposited to the sequence read archive (SRA) (BioProject ID: PRJNA1050576; BioSample accession number: SAMN38746387). Uncropped western blots are provided in the Supplementary Information. Additional data and materials that support the findings of the study can be found in the article and its supplementary material.

### Role of funders

The funders played no roles in the study design, data collection, data analysis, interpretation, or the writing of the manuscript.

## Results

### Identification of *CYP2D6* as a target gene for LOH-based therapy

**Fig. 2:**
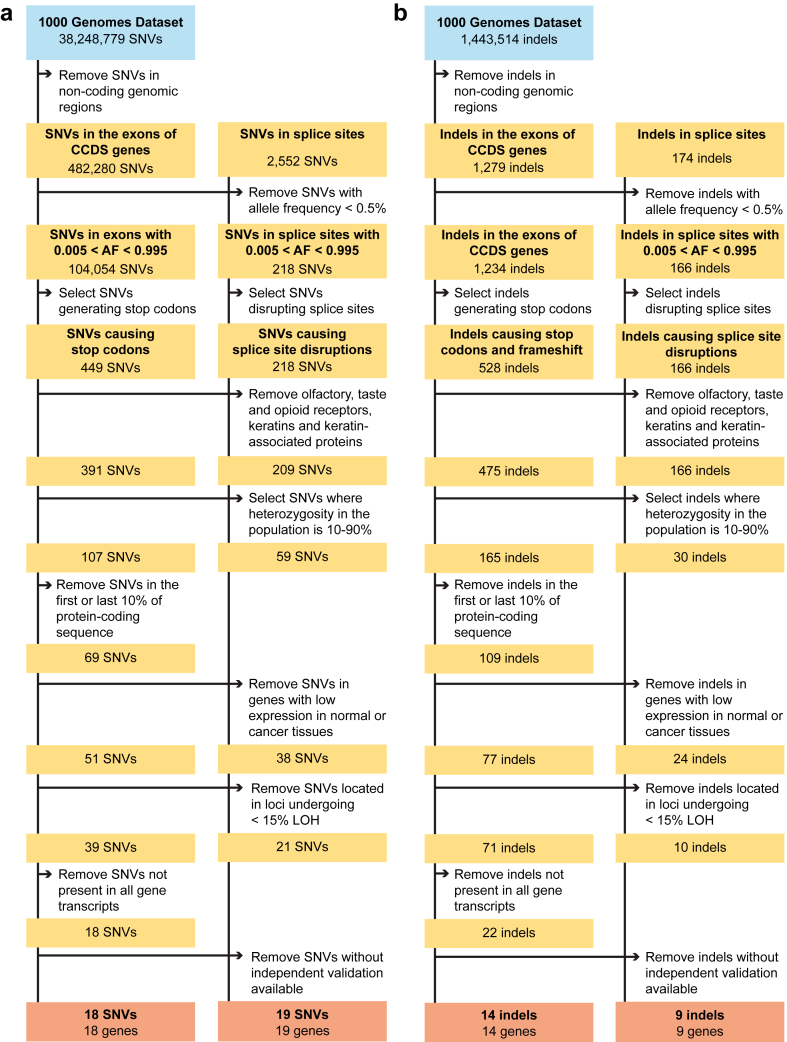
**Identification of *CYP2D******6*****as a target for LOH-directed therapy by analysis of common constitutional loss-of-function variants in the human genome**. Putative polymorphisms causing premature stop codons, frameshift or splice site mutations were identified by analysis of the 1000 Genomes Project (**a**) SNVs and (**b**) indels located near or within exons of the consensus coding genes (CCDS) gene set. The resulting loss-of-function polymorphisms of interest are present at 10–90% heterozygosity in the human population and in all alternative gene transcripts. This led to 361 polymorphisms in 325 genes for which a considerable number of heterozygous patients could be eligible and benefit from therapy. The 361 polymorphisms include 107 SNVs and 165 indels in the exons of CCDS genes, 59 SNVs and 30 indels in splice sites. These variants are additionally expressed in normal tissues and at least one common cancer type for which the frequency of undergoing loss of heterozygosity is at least 15%.

To identify potential candidate genes with prevalent LoF alleles and where LOH events observed in common cancer types could result in the exclusive retention of the LoF allele, our first objective was to compile a list of genes harbouring prevalent LoF variants in human populations. We mined the full Phase I 1000 Genomes dataset for SNVs causing premature stop codons, indels causing frameshift or truncation, and SNVs or indels causing splice site disruption. We discarded variants located in non-coding genomic regions and those with allele frequency <0.5%, thereby prioritizing alleles with a practically exploitable population prevalence.^22^ This removed ∼78.5% of alleles and retained 1361 potentially deleterious variants, consisting of 449 SNVs, 528 indels and 384 splice site alterations (Fig. 2 and Supplementary Table S4). Compared to a previous analysis of a subset of the 1000 Genomes dataset,^23^ a similar positional distribution of the variants within transcripts (with a predilection for the 3′ and 5’ ends of the transcripts) was observed (Supplementary Table S4). Interestingly, 60.5% of the alterations observed in this study were previously not described, and 42.4% of the variants observed in a previously published work^23^ were not observed here (Table 1).

**Table 1:** Comparison to previously identified mutations from preliminary analyses of the 1000 genomes dataset identifies known and new truncating polymorphisms.

LoF variant	Initial number reported by MacArthur et al.	In CCDS definitions of exons/splice sites	Overlap between our dataset and the reference set		LoF variants previously unreported
			Before AF filter	After AF filter	
Stop SNV	1111	809	580 (∼72%)	256 (∼32%)	193
Splice SNV	658	291	168 (∼58%)	86 (30%)	132
Frameshift indel	1040	421	241 (∼57%)	210 (∼50%)	355
Splice indel	0	0	0	0	166

The final sets of LoF variants reported by MacArthur et al.^23^ (second column) were imposed upon the exon/splice-junction definitions from the CCDS database, and only the variants within the defined borders were considered further (third column). The variants from the third column were matched towards the datasets from the current study before (fourth column) or after (fifth column) the filtering for AF (<0.005 or >0.995), and the percentage of overlap is shown in parenthesis. The last column shows the number of LoF variants from the current study, which are not reported by MacArthur et al.

Next, variants in genes unlikely to be exploitable in a cancer context, such as olfactory, taste and opioid receptors as well as keratins and keratin-associated proteins, were removed. Subsequently, we limited our selection to common variants, defined as polymorphisms with a heterozygosity prevalence ranging from 10% to 90% in the human population. This resulted in 361 polymorphisms in 325 genes. For SNVs causing premature stop codons and indels causing truncation and/or frameshift, variants predicted to disrupt less than 10% or over 90% of the protein-coding sequence were excluded, as such polymorphisms may lack functional impact on the translated protein because of alternative translation start sites or C-terminal truncations having no effect on the near full-length protein product. The remaining targets were filtered according to their expression profile, taking into consideration only those genes that were expressed in the normal and tumour tissues of interest. Next, we required the gene loci to be frequently lost through LOH in >15% of cases in at least one common cancer type.^13^ To enrich for functionally important variants at the transcriptional and protein level, we discarded polymorphisms not present in all known transcripts of each candidate gene. These selection criteria resulted in 18 SNVs in 18 different genes which generate premature stop codons, 22 indels in 22 different genes causing truncation or frameshift, and 31 splice site disruptions in 31 different genes caused by SNVs or indels. Altogether, we obtained 71 LoF variants in 71 genes which we proceeded to validate manually or by mining for independent support in alternative sequencing efforts. Independent support derived from HapMap, dbSNP and other genome sequencing efforts,^24^ was available for ∼85% of the LoF variants. As expected, the remaining unconfirmed polymorphisms were primarily indel mutations, of which ∼37% could not be validated by alternative resources. Therefore, the ultimate dataset comprised 60 LoF variants distributed across 60 distinct genes. These variants were subsequently categorized into five classes, each associated with predicted LoF mechanisms. The classes were (1) SNVs introducing a premature stop codon (16 genes); (2) SNVs disrupting a splice site (19 genes), (3) insertions or deletions causing a shift in the reading frame of a transcript (13 genes), (4) indels disrupting a splice site (9 genes), and (5) LoF variants involving two different mechanisms (3 genes) (Table 2). Together, these 60 genes are potential targets for LOH-directed therapeutic approaches.

**Table 2:** Genes with prevalent loss-of-function variants potentially exploitable in cancer therapies targeting loss of heterozygosity.

	Class 1: genes with prevalent LoF variants by a SNV-inducing premature stop codon			
	Gene and chromosomal location	Cancer types with LOH in ≥15% of cases^a^	LoF variant and percentage of heterozygotes	LoF variant percentage of the protein (%)
1	*SYNE2* 14q23.2	Colon: ∼15%, Kidney: ∼22%, Ovarian: ∼20% and Melanoma: ∼14%	rs2781377 (94% HET)	58.11
2	*UBE2NL* Xq27.3	Lung: ∼26%, Ovarian: ∼30%, Neuroblastoma: ∼24% and Neuroglial: ∼20%	rs237520 (48% HET)	57.79
3	*CLDN5* 22q11.21	Lung: ∼27%	rs885985 (44% HET)	12.21
4	*FUT2* 19q13.33	Lung: ∼25% and Ovarian: ∼18%	rs601338 (35% HET)	44.77
5	*C17orf77* 17q25.1	Lung: ∼17% and Ovarian: ∼24%	rs545652 (26% HET)	85.19
6	*ARMS2* 10q26.13	Lung: ∼22%, Ovarian: ∼18%, Melanoma: ∼23% and Neuroblastoma: ∼15%	rs2736911 (22% HET)	35.51
7	*MROH2B* 5p13.1	Lung: ∼16% and Ovarian: ∼15%	rs1023840 (22% HET)	12.05
8	*PRB4* 12p13.2	Lung: ∼20%, Ovarian: ∼17% and Melanoma: ∼15%	rs12829245 (18% HET)	21.91
9	*ARMC3* 10p12.2	Lung: ∼24%, Ovarian: ∼23%, Melanoma: ∼20% and Neuroglial: ∼31%	rs190663005 (17% HET)	75.61
10	*HLA-DQB1* 6p21.32	Lung: ∼17% and Ovarian: ∼15%	rs1130385 (17% HET)	40.46
11	*AP3M1* 10q22.2	Lung: ∼22%, Melanoma: ∼20% and Neuroglial: ∼30%	rs190136804 (15% HET)	61.72
12	*EIF3CL* 16p11.2	Lung: ∼20% and Ovarian: ∼18%	rs201261076 (15% HET)	63.79
13	*PRAMEF2* 1p36.21	Breast: ∼19%, Colon: ∼18%, Lung: ∼26%, Ovarian: ∼19%, Melanoma: ∼18% and Neuroblastoma: ∼50%	rs75411676 (15% HET)	44.51
14	*SPERT* 13q14.13	Lung: ∼29% and Ovarian: ∼20%	rs79707842 (14% HET)	60.68
15	*OBSCN* 1q42.13	Lung: ∼20% and Ovarian: ∼20%	rs3795786 (11% HET)	42.64
16	*ZNF860* 3p23	Kidney: ∼49%, Lung: ∼44%, Ovarian: ∼18%, Melanoma: ∼29% and Neuroblastoma: ∼15%	rs4639011 (11% HET)	22.63

	Class 2: genes with prevalent LoF variants through a SNV-disrupting splice site		
	Gene and chromosomal location	Cancer types with LOH in ≥15% of cases^a^	LoF variant and percentage of heterozygotes
1	*CCDC182* 17q22	Lung: ∼17% and Ovarian: ∼21%	rs12451748 (49% HET)
2	*HTR3D* 3q27.1	Ovarian: ∼18% and Melanoma: ∼28%	rs6443930 (47% HET)
3	*TOR1AIP1* 1q25.2	Lung: ∼16%	rs2245425 (44% HET)
4	*ZNF419* 19q13.43	Lung: ∼25% and Ovarian: ∼18%	rs2074071 (43% HET)
5	EMR1 19p13.3	Lung: ∼22% and Ovarian: ∼15%	rs330880 (42% HET)
6	*GSDMB* 17q12	Lung: ∼18% and Ovarian: ∼21%	rs11078928 (42% HET)
7	*OAS1* 12q24.13	Melanoma: ∼16%	rs10774671 (41% HET)
8	*TMPRSS4* 11q23.3	Ovarian: ∼18%, Melanoma: ∼19% and Neuroblastoma: ∼19%	rs2276122 (35% HET)
9	*TBC1D31* 8q24.13	Lung: ∼21% and Ovarian: ∼21%	rs10101626 (32% HET)
10	*C14orf105* 14q22.3	Kidney: ∼22%, Lung: ∼21% and Ovarian: ∼21%	rs1152522 (30% HET)
11	*C14orf159* 14q32.11	Lung: ∼22% and Ovarian: ∼20%	rs4900072 (29% HET)
12	*NPHP4* 1p36.31	Breast: ∼20%, Colon: ∼18%, Lung: ∼26%, Ovarian: ∼19%, Melanoma: ∼18% and Neuroblastoma: ∼50%	rs1287637 (28% HET)
13	*TNK1* 17p13.1	Colon: ∼31%, Lung: ∼35%, Ovarian: ∼24% and Melanoma: ∼15%	rs7220814 (17% HET)
14	*CYP2D6* 22q13.2	Lung: ∼28%, Ovarian: ∼25%, Neuroblastoma: ∼18% and Neuroglial: ∼22%	rs3892097 (16% HET)
15	*MPP2* 17q21.31	Lung: ∼17% and Ovarian: ∼21%	rs231518 (15% HET)
16	*EFCAB13* 17q21.32	Lung: ∼17% and Ovarian: ∼21%	rs76299620 (11% HET)
17	*SLC22A14* 3p22.2	Kidney: ∼49%, Lung: ∼44%, Ovarian: ∼18%, Head/neck: ∼19%, Melanoma: ∼29% and Neuroblastoma: ∼15%	rs753331 (11% HET)
18	*CES5A* 16q12.2	Lung: ∼17%, Ovarian: ∼15% and Melanoma: ∼21%	rs72810507 (10% HET)
19	*ENDOV* 17q25.3	Lung: ∼17% and Ovarian: ∼21%	rs41298712 (10% HET)

	Class 3: genes with prevalent LoF variants through an indel-causing shift in the reading frame of a transcript			
	Gene and chromosomal location	Cancer types with LOH in ≥15% of cases^a^	LoF variant and percentage of heterozygotes	Percentage of the protein occurring the LoF variant (%)
1	*ZNF880* 19q13.41	Lung: ∼25% and Ovarian: ∼18%	rs34470614 (43% HET)	18.37
2	*C10orf113* 10p12.31	Lung: ∼22%, Ovarian: ∼15%, Melanoma: ∼20%, Neuroblastoma: ∼15% and Neuroglial: ∼30%	rs72102767 (35% HET)	40.51
3	*EBLN2* 3p13	Kidney: ∼48%, Lung: ∼40%, Ovarian: ∼18%, Head/neck: ∼19% and Melanoma: ∼28%	NT73111480chr3 (28% HET)	30.88
4	*GLT6D1* 19q34.3	Lung: ∼31% and Ovarian: ∼18%	rs34217442 (27% HET)	51.81
5	*CD200R1L* 3q13.2	Kidney: ∼16% and Melanoma: ∼28%	rs58161637 (27% HET)	72.8
6	*C12orf60* 12p12.3	Ovarian: ∼15%	rs139293175 (26% HET)	75.1
7	*RAI1* 17p11.2	Colon: ∼29%, Lung: ∼29% and Ovarian: ∼23%	rs35068024 (23% HET)	14.69
8	*AKAP3* 12p13.32	Lung: ∼20%, Ovarian: ∼17% and Melanoma: ∼15%	rs67512580 (22% HET)	82.06
9	*ZNF681* 19p12	Lung: ∼22% and Ovarian: ∼15%	rs61397759 (22% HET)	31.47
10	*PRR25* 16p13.3	Lung: ∼21%, Ovarian: ∼16% and Melanoma: ∼21%	rs138733834 (21% HET)	58.71
11	*ZNF812* 19p13.2	Lung: ∼24% and Ovarian: ∼19%	rs112014279 (18% HET)	53.96
12	*SLFN12L* 17q12	Lung: ∼18% and Ovarian: ∼21%	rs143471015 (12% HET)	22.11
13	*MUC22* 6p21.33	Lung: ∼17% and Ovarian: ∼15%	rs112064513 (11% HET)	35.93

	Class 4: genes with prevalent LoF variants through an indel-disrupting splice site		
	Gene and chromosomal location	Cancer types with LOH in ≥15% of cases^a^	LoF variant and percentage of heterozygotes
1	*NEK3* 13q14.3	Lung: ∼29% and Ovarian: ∼20%	rs3837575 (43% HET)
2	*ATG2B* 14q32.2	Lung: ∼22% and Ovarian: ∼20%	rs34296665 (42% HET)
3	*SPATA6L* 9p24.1	Lung: ∼38%, Ovarian: ∼18%, Melanoma: ∼18% and Neuroglial: ∼19%	rs34533529 (38% HET)
4	*NFKBIZ* 3q12.3	Kidney: ∼16% and Melanoma: ∼28%	rs3217713 (31% HET)
5	*MPRIP* 17p11.2	Colon: ∼29%, Lung: ∼29% and Ovarian: ∼23%	rs3215213 (29% HET)
6	*TMPRSS3* 21q22.3	Lung: ∼26% and Ovarian: ∼24%	rs56283966 (25% HET)
7	*A2M* 12p13.31	Lung: ∼20%, Ovarian: ∼17% and Melanoma: ∼15%	rs3832852 (23% HET)
8	*PDZRN3* 3p13	Kidney: ∼48%, Lung: ∼40%, Ovarian: ∼18%, Head/neck: ∼19% and Melanoma: ∼28%	rs200593963 (21% HET)
9	*SLC3A1* 2p21	Ovarian: ∼15%	rs61179824 (17% HET)

	Class 5: genes with prevalent LoF variants involving two predicted mechanisms				
	Gene and chromosomal location	Cancer types with LOH in ≥15% of cases^a^	LoF variant and percentage of heterozygotes	LoF predicted mechanism	Percentage of the protein occurring the LoF variant (%)
1	*GSTT2* 22q11.23	Lung: ∼27%	rs201176441 (29% HET)	SNV introducing a premature stop codon	80.33
			rs76498342 (33% HET)	SNV disrupting a splice site	–
2	*GSTT2B* 22q11.23	Lung: ∼27%	rs200376763 (26% HET)	SNV introducing a premature stop codon	80.33
			rs181983734 (25% HET)	SNV disrupting a splice site	–
3	*CYP4B1* Cytochrome 11p33	Breast: ∼19%, Colon: ∼18%, Lung: ∼26%, Ovarian: ∼19%, Melanoma: ∼18% and Neuroblastoma: ∼50%	rs3215983 (25% HET)	Indel causing a shift in the reading frame of a transcript	57.53
			rs3215983 (25% HET)	Indel disrupting a splice site	–

Sixty loss-of-function variants in 60 potential target genes causing likely loss of gene function through premature stop codons, frameshifts or splice site disruption. Information on protein function was obtained from UniProt (http://www.uniprot.org). Gene expression levels and LOH frequency in common cancers was analyzed to obtain a set of candidate genes expressed in at least one common form of cancer and lost in ≥15% of patient tumors. Protein function data was obtained from the UniProt database (http://www.uniprot.org).

a The fraction of tumors with LOH at the target locus was estimated from Mertens et al., 1997 for the following forms of cancer: breast, colon, kidney, lung, ovarian, head and neck, malignant melanoma, neuroblastoma and neuroglial tumors.^13^

Knowledge on protein function was available for two-thirds of the candidate genes and revealed genes regulating cell functions (i.e., transcriptional regulation, apoptosis, cell growth and proliferation) or playing a role in metabolic pathways. A subset of these polymorphisms had previously been associated with disease (Supplementary Table S2). The variant rs601338 in *FUT2* has been associated with resistance to Norwalk virus infection,^25^ rs10774671 in *OAS1* with diabetes type 1 susceptibility,^26^ rs3892097 in *CYP2D6* with poor metabolism of debrisoquine,^27^ and rs3832852 in *A2M* with the pathogenesis of Alzheimer's disease.^28^ Additionally, ∼18% of the splice site variants had been linked to the development of cancer and other complex diseases such as asthma and autoimmune disorders in independent studies. Additionally, after assessing variants of these 60 genes in the Genome Aggregation Database (gnomAD), variants in 51 of these genes, including the LOH variant rs3892097 in *CYP2D6*, remained (Supplementary Table S5 and Supplementary data 1).

The cytochrome P450 oxidase family member CYP2D6 is known to metabolize ∼25% of drugs currently in clinical use by adding or removing specific functional groups through processes such as hydroxylation, demethylation, and dealkylation.^29^ The gene encoding CYP2D6 is located on 22q13.2, a locus undergoing LOH in >30% of hepatocellular carcinomas^30^ and >15% of neuroblastoma, glioblastoma, neuroglial, lung and ovarian tumours.^31^^,^^32^ Among its many known alleles, the rs3892097 variant disrupts the recognition of the acceptor splice site in exon 4, resulting in the generation of an aberrant transcript that produces a truncated product.^33^ This non-functional allele, also termed *CYP2D6∗4*, lacks activity and results in a poor metabolizer phenotype.^34^ The allele frequency for *CYP2D6∗4* is highly variable, being rare in Chinese (∼1%)^35^ and African populations (∼4%)^36^ but common in Caucasians (20–25%).^37^ Due to its well-established role in pharmacogenetics, significant impact on drug effects and toxicity, and the relatively high frequency of LOH events, we concentrated our efforts on *CYP2D6* and its LoF variant rs3892097.

### Identification of anti-cancer drugs with CYP2D6 dependent activity

**Fig. 3:**
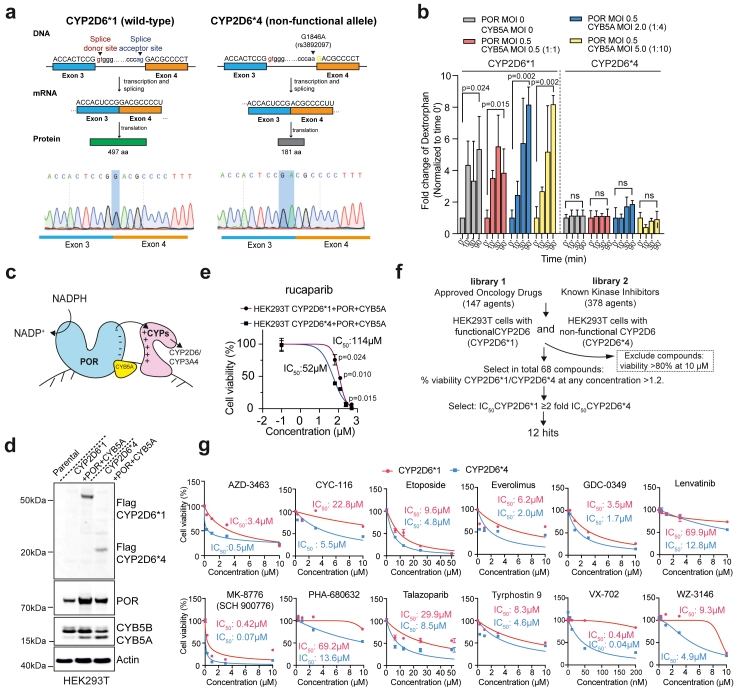
**Establishment of cell models expressing functional CYP2D6∗1 or loss-of-function CYP2D6∗4 alleles**. **a**. Upper panel: as part of our drug discovery toolset, we generated models expressing either the wild-type *CYP2D6* or the loss-of-function *CYP2D6* allele associated with rs3892097 (*CYP2D6∗4*). *CYP2D6∗1* allele encodes the wild-type sequence, producing a transcript that is translated into a 497 amino acid (aa) protein (green). In contrast, the *CYP2D6∗4* variant creates a cryptic acceptor site at the start of exon 4 (yellow), creating a frameshift when transcription and splicing occurs. The resulting aberrant transcript is missing 1 bp in exon 4 and is translated into a truncated peptide of 181 amino acids (grey). Lower panel: genotyping of the generated clones confirmed that they encode either the wild-type *CYP2D6∗1* or the *CYP2D6∗4* allele resulting from the 1846 G > A splice variant. The coding part of exon 3 (blue) and exon 4 (orange) is shown. **b.** Detection of CYP2D6 catalytic activity in HEK293T cells overexpressing wild-type or LoF *CYP2D6* alleles, and transiently overexpressing cofactors POR and CYB5A. The formation of dextrorphan was measured by LC-MS/MS at indicated time points after incubation with 10 μM of the CYP2D6 specific substrate dextromethorphan. One representative experiment with three technical replicates is shown (mean ± SD, POR MOI:0/CYB5A MOI:0, 90′ incubation p = 0.024; POR MOI:0.5/CYB5A MOI:0.5, 90′ incubation p = 0.015; POR MOI:0.5/CYB5A MOI:2.0, 90′ incubation p = 0.002; POR MOI:0.5/CYB5A MOI:5.0, 90′ incubation p = 0.002; unpaired t-test, Two-stage step-up (Benjamini, Krieger, and Yekutieli)). **c.** Schematic of POR and CYB5A in relation to the CYP family CYP2D6 and CYP3A4. POR donates electrons to CYPs, which in turn donates them to molecular oxygen as the terminal electron acceptor thereby mediating catalysis. In some cases, CYB5A facilitates interaction of POR with CYPs, and may act as an alternative donor for the second, but not the first, electron in the P450 cycle; thus, the action of POR is essential for all CYPs.^39^**d.** Confirmation of HEK293T CYP2D6∗^1+POR + B5A^ and CYP2D6∗^4+POR + B5A^ cell models. Expression of FLAG-tagged wild-type CYP2D6∗1 and LoF CYP2D6∗4, POR and CYB5A was confirmed by immunoblotting with actin as loading control. **e.** Dose response to the CYP2D6 specific substrate rucaparib in HEK293T CYP2D6∗^1+POR + B5A^ and CYP2D6∗^4+POR + B5A^ cells. One representative experiment with three technical replicates is shown (mean ± SD, rucaparib 65 μM, p = 0.024; rucaparib 130 μM, p = 0.010; unpaired t-test, Two-stage step-up (Benjamini, Krieger, and Yekutieli)). **f.** Workflow of the cell-based drug screen. A total of 525 compounds from the approved oncology drug set and known kinase inhibitors set were assessed in a cell-based screen to find compounds that exhibited selective toxicity to either HEK293T cells with CYP2D6∗^1+POR + B5A^ (thereafter HEK293T CYP2D6∗1 in the figure) or CYP2D6∗^4+POR + B5A^ (thereafter HEK293T CYP2D6∗4 in the figure). **g.** Dose response to 12 identified hit compounds in the HEK293T cell model. Name of 12 compounds: AZD-3463, CYC-16, etoposide, everolimus, GDC-0349, lenvatinib, MK-8776, PHA-680632, talazoparib, tyrphostin 9, VX-702 and WZ-3146. The half-maximal inhibitory concentration (IC_50_) was calculated using Dose–Response inhibition (inhibition vs normalized response).

To identify clinically used anticancer drugs whose effects are influenced by CYP2D6 activity, we established cancer cell models proficient or deficient in CYP2D6 activity by introducing the wild-type *CYP2D6∗1* and LoF *CYP2D6∗4* variant containing the splice site substitution G1846A (rs3892097), which creates a new acceptor site one base downstream of exon 4. We designed a lentiviral insert with a transcript lacking the first coding base of *CYP2D6* exon 4, as this is the most likely aberrant transcript resulting from the splice site variant.^38^ Next, we confirmed transcript expression from wild-type *CYP2D6∗1* and the LoF *CYP2D6∗4* variant. Genotyping of the generated clones confirmed that they encode either the wild-type *CYP2D6∗1* allele or the LoF variant *CYP2D6∗4* allele transcripts resulting from the 1846 G > A splice variant (Fig. 3a, bottom). Cells overexpressing the wild-type *CYP2D6∗1* allele will produce a full-length functional CYP2D6 enzyme containing 497 amino acids, but cells overexpressing the LoF *CYP2D6∗4* variant with the splice G1846A site will produce a truncated non-functional CYP2D6 enzyme containing only 181 amino acids (Fig. 3a, Upper). Since HEK293T cells do not endogenously express CYPs (proteinatlas and^40^), which minimizes the potential interference from other drug metabolic enzymes,^40^ we first stably overexpressed the wild-type or LoF CYP2D6 proteins in HEK293T cells (Supplementary Figure S1a). Next, the catalytic activity of CYP2D6 was assessed by measuring the formation of dextrorphan which is generated from the CYP2D6-specific substrate dextromethorphan. Dextrorphan formation increased 5-fold after 90min incubation with dextromethorphan in wild-type CYP2D6 but not in LoF CYP2D6 expressing cells (dextrorphan formation at 90′ vs 0’, unpaired t-test, Fig. 3b, grey bar), supporting that the LoF CYP2D6 protein lacks catalytic activity.

The electron donors cytochrome P450 oxidoreductase (POR) and cytochrome b5 (CYB5A) are essential components regulating the activities of cytochrome P450 oxidase enzymes and have been used in HEK293T cells to enhance CYP3A4 activity.^41^ Since CYP2D6 and CYP3A4 share the same electron donors (Fig. 3c and^42^), we transiently overexpressed POR and CYB5A in HEK293T cells overexpressing wild-type or LoF CYP2D6 (Supplementary Figure S1b and 1c) and determined its activity. With increasing levels of POR and CYB5A, the formation of dextrorphan was up to ∼8-fold higher in wild-type CYP2D6∗1 overexpressing cells, but not in LoF CYP2D6∗4 overexpressing cells (dextrorphan formation at 90′ vs 0’, unpaired t-test, Fig. 3b, blue and yellow bar). Since POR and CYB5A could further promote the catalytic activity of CYP2D6, we established a stable co-expression HEK293T cell model overexpressing wild-type CYP2D6∗1 (hereafter CYP2D6∗^1+POR+B5A^) or LoF CYP2D6∗4 (hereafter CYP2D6∗^4+POR+B5A^). The HEK293T CYP2D6∗^1+POR+CYB5A^ or CYP2D6∗^4+POR+CYB5A^ cells were confirmed by immunoblotting (Fig. 3d) and had no significant difference in growth rate (Supplementary Figure S1d).

CYP2D6 primarily metabolizes lipophilic compounds, including certain antidepressants, antipsychotics, antiarrhythmics, antiemetics, β-adrenergic receptor antagonists, and opioids. The processing of anticancer drugs by CYP2D6 has not been systematically studied,^43^ but CYP2D6 is known to metabolize the poly (ADP-ribose) polymerase (PARP) inhibitor rucaparib,^44^ suggesting that cells lacking CYP2D6 should exhibit increased sensitivity to rucaparib. Compared to HEK293T CYP2D6∗^1+POR+B5A^, CYP2D6∗^4+POR+B5A^ cells exhibited increased sensitivity to rucaparib (IC_50_ 114 μM vs 52 μM, [Inhibitor] vs. normalized response, Fig. 3e). Next, we sought to identify other anticancer drugs already in clinical use or in clinical trials that display *CYP2D6* allele-specific cytotoxicity. To do so, we performed drug screening using the HEK293T cell model. A total of 525 approved cancer drugs and kinase inhibitors were evaluated by dose–response screening (Fig. 3f). This screen identified 12 drugs, AZD-3463 (ALK/IGF1R inhibitor), CYC-116 and PHA-680632 (aurora kinase inhibitor), etoposide (topoisomerase II inhibitor), everolimus and GDC-0349 (mTOR kinase inhibitor), lenvatinib, tyrphostin 9 and WZ-3146 (GFRs inhibitor), talazoparib (PARP inhibitor), MK-8776 (checkpoint kinase1 inhibitor) and VX-702 (p38 MAP kinase inhibitor), having ≥2-fold higher potency (IC_50_) in HEK293T CYP2D6∗^4+POR+B5A^ cells (Fig. 3g, Supplementary Figure S1e). Taken together, 2.3% of the evaluated compounds displayed CYP2D6 dependent toxicity.

### Validating hit candidates in hepatocellular carcinoma cells with deficient CYP2D6 expression

**Fig. 4:**
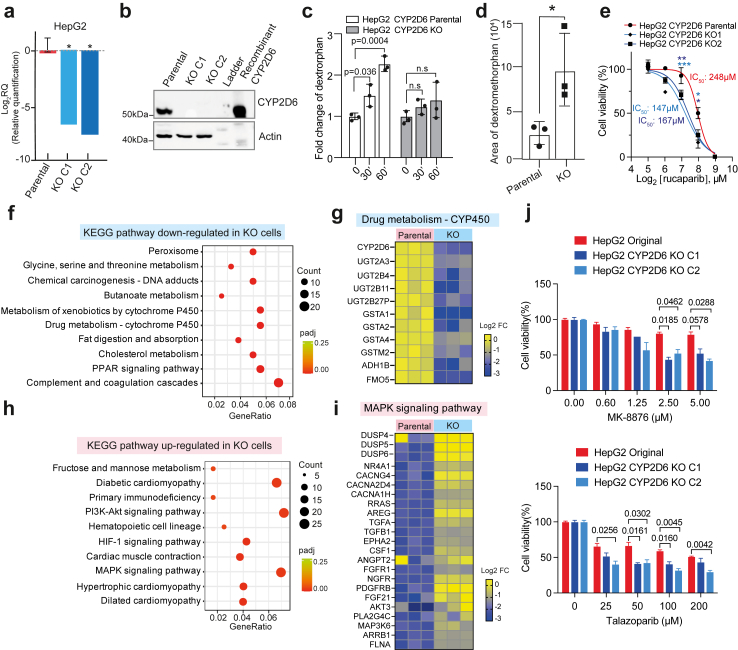
**Talazoparib and MK-8776 exhibit CYP2D6 dependent cytotoxicity in hepatocellular carcinoma cells**. **a.** Relative expression of CYP2D6 in parental HepG2 cells and two CRISPR/Cas9 *CYP2D6* KO clones was determined by qPCR. ∗p < 0.05 by two-tailed t-test. RQ: relative quantification. **b.** Expression levels of endogenous CYP2D6 protein was determined in HepG2 parental and *CYP2D6* KO clones by immunoblotting using actin as loading control. Recombinant CYP2D6 is pure CYP2D6 protein thus no actin was detected. **c.** Catalytic activity of CYP2D6 in HepG2 parental and *CYP2D6* KO cells. The formation of dextrorphan was measured by LC-MS/MS at indicated time points after incubation with 10 μM of the CYP2D6 specific substrate dextromethorphan. One representative experiment with three technical replicates is shown (mean ± SD, HepG2 parental 30′ vs 0′ incubation, p = 0.036; HepG2 parental 60′ vs 0′ incubation, p = 0.0004, unpaired t-test, Two-stage step-up (Benjamini, Krieger, and Yekutieli)). **d.** The total amount of the CYP2D6-specific substrate dextromethorphan in the cell pellet was determined by LC-MS/MS after 90min incubation with 10 μM dextromethorphan. One representative experiment with three technical replicates is shown (mean ± SD, ∗p < 0.05 by two-tailed t-test). **e.** Dose–response to rucaparib in HepG2 parental and *CYP2D6* KO clones. One representative experiment with three technical replicates is shown (mean ± SD, ∗p < 0.05, ∗∗p < 0.01 and ∗∗∗p < 0.001, unpaired t-test, Two-stage step-up (Benjamini, Krieger, and Yekutieli)). **f.** The top 10 down-regulated KEGG signalling pathways in HepG2 *CYP2D6* KO cells compared to HepG2 parental cells (full list Supplementary Table S6). **g.** The enriched genes in the KEGG pathway of drug metabolsim-CYP450. **h.** The top 10 up-regulated KEGG signalling pathways in HepG2 *CYP2D6* KO cells compared to HepG2 parental cells (full list Supplementary Table S7). **i.** The enriched genes in the MAPK signalling pathway. **j.** Dose–response of talazoparib and MK-8776 in HepG2 parental and *CYP2D6* KO clones. Cell viability of KO clone 1 vs parental, and KO clone 2 vs parental, unpaired t-test, Two-stage step-up (Benjamini, Krieger, and Yekutieli), only show p-values <0.05).

Hepatocellular carcinoma HepG2 cells are inherently resistant to many chemotherapeutic drugs,^45^^,^^46^ thus representing a challenging model for drug screening. Even so, the HepG2 cells are among the few available options suitable for hit validation since they (1) are homozygous for the wild-type *CYP2D6∗1* allele, thus suitable for CRISPR editing; (2) express the cofactors required for cytochrome P450 activity^47^; (3) express other CYPs allowing determination of specificity. We hypothesized that HepG2 cells lacking functional CYP2D6 should be more sensitive to our discovered hit compounds. To validate this, we established two CRISPR/Cas9 *CYP2D6* knockout clones (KO C1 and C2) in HepG2 cells and showed that they lacked endogenous expression of CYP2D6 (Fig. 4a and b). We observed a higher increase of dextrorphan formation in HepG2 parental cells than in *CYP2D6* KO clones (dextrorphan formation at 30′ vs 0′, and 60′ vs 0′, unpaired t-test, Fig. 4c). Furthermore, compared to HepG2 parental cells, a ∼4-fold accumulation of dextromethorphan was observed in *CYP2D6* KO cells after 90 min incubation, suggesting that *CYP2D6* KO cells take up but are unable to metabolize dextromethorphan (dextrorphan formation at 90′ vs 0’, unpaired t-test, Fig. 4d, Supplementary Figure S2a). Moreover, *CYP2D6* KO cells exhibited greater sensitivity to rucaparib (Fig. 4e), a known substrate of CYP2D6, thereby validating the reliability of the HepG2 cell model for hit validation.

To gain a deeper understanding of how loss of CYP2D6 expression affects cellular function, we performed RNA-SEQ analysis and compared differential KEGG pathway analysis between HepG2 *CYP2D6* parental and KO cells. Interestingly, the top downregulated pathways in HepG2 *CYP2D6* KO cells included cholesterol metabolism (padj = 0.0001), fat digestion and absorption (padj = 0.0005), and CYP450-mediated drug metabolism and peroxisome (padj = 0.0008) (Fig. 4f and g, Supplementary Figure S2b and Supplementary Table S6), which have been associated with the activity of CYP2D6.^48^, ^49^, ^50^ The pathways most significantly upregulated in *CYP2D6* KO cells included those associated with cardiomyopathy (padj = 0.0016), MAPK signalling (padj = 0.0059), and the PI3K-Akt signalling pathway (padj = 0.0159) (Fig. 4h and i, Supplementary Figure S2c and 2d and Supplementary Table S7), which has also been reported previously,^50^^,^^51^ suggesting that altered CYP2D6 activity or expression level may not only affect drug metabolism but also alter cellular function. Moreover, several growth factor ligands and receptors (PDGFRB, NGFR, TGFA, FGFR1 and FGF21) were identified as jointly upregulated genes in the MAPK signalling and the PI3K-Akt signalling pathways (Supplementary Figure S2e). Next, we tested the 12 hit compounds selected from the HEK293T cell system using the HepG2 cell model. AZD-3463, CYC-116, etoposide, tyrphostin 9, VX-702 and WZ-3146 were excluded due to a lack of CYP2D6 selective toxicity in HepG2 cells; lenvatinib was excluded because of its IC_50_ >50 μM. Everolimus, PHA-680632 and GDC-0349 were excluded because the difference in IC_50_ was less than two-fold. The drugs MK-8776 and talazoparib were retained as hits (cell viability of KO clone 1 vs parental, and KO clone 2 vs parental, unpaired t-test, Fig. 4j, Supplementary Figure S2f and g).

In a similar vein, we hypothesized that cells overexpressing functional CYP2D6 should be more tolerant to the hit candidates compared to those overexpressing LoF CYP2D6. HepG2 cells were therefore stably transduced with lentiviral particles encoding functional CYP2D6∗1 or LoF CYP2D6∗4 (Supplementary Figure S3a and b). A kinetic analysis of CYP2D6 catalytic activity revealed that dextrorphan formation was ∼8-fold higher in CYP2D6∗1 overexpressing HepG2 cells, non-functional CYP2D6∗4 clones showed the same level of dextrorphan formation as parental HepG2 cells after 90mins incubation with dextromethorphan (dextrorphan formation at 90′ vs 0’, unpaired t-test, Supplementary Figure S3c). Considering the significant endogenous CYP2D6 expression in HepG2 cells (proteinatlas.org) and the similar level of dextrorphan production observed in parental HepG2 and CYP2D6∗4 cells (unpaired t-test, Supplementary Figure S3d), it is evident that also the cells overexpressing the LoF CYP2D6∗4 still maintain drug detoxification ability. Nevertheless, talazoparib and MK-8776 showed consistent specific cytotoxicity against HepG2 cells overexpressing LoF CYP2D6∗4 (Supplementary Figure S3e).

Apart from CYP2D6, the CYP3A subfamily, specifically CYP3A4, is the most abundant CYP member in the human body and metabolizes >30% of clinically used drugs.^52^ We therefore asked if CYP3A4 activity would affect the effect of talazoparib or MK-8776 in HepG2 CYP3A4 overexpressing cells with∼10-fold higher CYP3A4 activity compared to HepG2 parental cells. Ketoconazole, a specific CYP3A4 inhibitor, inhibited CYP3A4 activity in HepG2 CYP3A4 overexpressing cells (Supplementary Figure S3f). In the presence or absence of ketoconazole, HepG2 parental and CYP3A4 overexpressing cells treated with talazoparib or MK-8776 did not display big differences in survival (Supplementary Figure S3g), demonstrating that CYP3A4 activity does not significantly alter the effect of these drugs. Similarly, CYP1A2 and CYP2C9 activity did not impact the cytotoxic effects of talazoparib and MK-8776 (Supplementary Figure S3h–k). Taken together, the cytotoxicity of talazoparib and MK-8776 in HepG2 cells was dependent on CYP2D6, but not on the activity of the other abundant CYP members.

### Talazoparib exhibits cytotoxicity against cells deficient in CYP2D6 activity in 3D culture models and human liver cancer-derived organoids

**Fig. 5:**
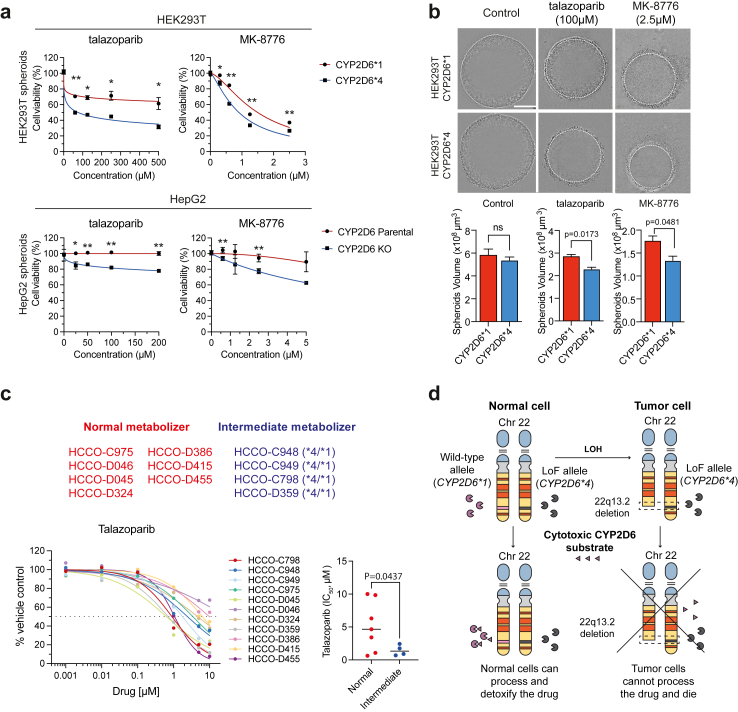
**Talazoparib exhibits cytotoxicity against cells with deficient CYP2D6 activity in 3D culture models and hepatocellular carcinoma organoids**. **a.** Dose response to talazoparib and MK-8776 in spheroids formed by HEK293T CYP2D6∗^1+POR + B5A^ and CYP2D6∗^4+POR + B5A^ cells, or in spheroids formed by HepG2 parental and *CYP2D6* KO cells. One representative experiment with three technical replicates is shown (mean ± SD, ∗p < 0.05, ∗∗p < 0.01, unpaired t-test, Two-stage step-up (Benjamini, Krieger, and Yekutieli)). **b.** Representative images for HEK293T CYP2D6∗^1+POR + B5A^ and CYP2D6∗^4+POR + B5A^ cells were taken by Incucyte after 72 h incubation with indicated drugs. Diameters were measured and spheroid volumes were calculated using the formula V = (4/3) ∗π ∗Rˆ3, where π is approximately 3.14, and R represents the spheroid radius. One representative experiment with three technical replicates is shown (mean ± SD, two-tailed t-test, only show p-values <0.05). Bar scale = 300 μm. **c.** Dose response of human HCCOs to talazoparib. HCCO-C975, HCCO-D045, HCCO-D046, HCCO-D324, HCCO-D386, HCCO-D415, and HCCO-D455 were determined as normal metabolizers; HCCO-C798, HCCO-C948, HCCO-C949, and HCCO-D359 were determined as intermediate metabolizers. ∗p < 0.05, using unpaired t test with Welch's correction. **d**. Targeting *CYP2D6* allelic loss in tumors. *CYP2D6* is located on chromosome 22 (p-arm, blue; centromere, grey; q-arm, yellow), which is acrocentric (shown by the two black lines). Eligible patients are heterozygous for the wild-type *CYP2D6* allele (*CYP2D6∗1*, pink) and the LoF allele (*CYP2D6∗4*, dark grey). Throughout cancer progression, tumor cells undergo LOH and may lose the wild-type *CYP2D6* allele. Treatment with a cytotoxic CYP2D6 substrate (pink) will result in selective killing of tumor cells, as they cannot process the drug and die. Normal cells which still express CYP2D6 are able to detoxify the drug and survive.

Three-dimensional cell culture models more closely mimic tumor properties than monolayer cultures in terms of growth kinetics and metabolic rates.^53^ Moreover, cancer cells are in general more resistant to anticancer agents when grown as 3-dimensional spheroids rather than as monolayer cultures.^54^ We therefore evaluated the effect of talazoparib and MK-8776 in 3D culture models. We treated HEK293T overexpressing wild-type CYP2D6 or LoF CYP2D6, and HepG2 parental and *CYP2D6* KO spheroids with increasing concentrations of talazoparib and MK-8776, and measured cell viability after 72 h. Spheroids with LoF CYP2D6 exhibited higher sensitivity to talazoparib and MK-8776 (unpaired t-test, ∗p < 0.5, ∗∗p < 0.01, Fig. 5a). On spheroid microplates, HEK293T cells formed neat spheroids with sharp edges, whereas HepG2 cells could only form a 3D structure (Supplementary Figure S4a). We further calculated the size of HEK293T cells formed spheroids, a significant decrease in volume after treatment with talazoparib or MK-8776 was observed in HEK293T LoF CYP2D6 spheroids as compared to wild-type CYP2D6 spheroids (two-tailed t-test, Fig. 5b).

Considering that talazoparib is already used in the clinical setting, and consistently demonstrated CYP2D6-dependent cytotoxicity in both HEK293T and HepG2 spheroids, we proceeded to culture and treat human hepatocellular cancer organoids from 11 patients with talazoparib (two-tailed t-test, Fig. 5c). By genotyping CYP2D6 in each sample, we attempted to establish an association between predicted CYP2D6 catalytic activity and response to drug treatment (Supplementary Table S3). In general, tumour organoids identified as CYP2D6 intermediate metabolizers were more sensitive to talazoparib than normal metabolizers (Fig. 5d), supporting its potential as therapy for heterozygous patients only retaining the LoF allele in their tumors.

## Discussion

In 2020, LOH of essential genes was shown to constitute a significant category of non-driver cancer vulnerabilities when 5664 variants in 1278 essential genes that undergo LOH in cancer were identified and allele-specific inactivation of two such genes (PRIM1 and EXOSC8) was shown to be lethal to cancer cells.^2^ In our earlier research, we reported that non-driver cancer vulnerabilities resulting from LOH can be targeted using small molecules.^3^ To extend this concept, we here identified genes frequently lost in common cancers that have prevalent constitutional LoF alleles in the population. We ultimately selected the truncating rs3892097 variant of *CYP2D6* because (i) 22q13.2 undergoes LOH in >30% of hepatocellular carcinomas and >15% of cases of several tumor types, including neuroblastoma, glioblastoma, neuroglial, lung, and ovarian tumors^31^^,^^32^; (ii) CYP2D6 is a well-studied drug metabolic enzyme and its typical substrates are mainly antidepressants, antipsychotics, antiarrhythmic drugs, antiemetics and opioids, but there is limited knowledge of its metabolism of cancer drugs; and (iii) the carrier frequency of the LoF allele *CYP2D6∗4* is high among Caucasians, ranging from 20% to 25%.^37^^,^^55^ Currently, over 135 unique *CYP2D6* alleles, resulting from SNVs, indels and gene rearrangements, have been reported by the Pharmacogene Variation Consortium (PharmVar). The resulting protein variants are classified by activity level and enable the sorting of individuals within a population into poor, intermediate, normal (extensive), or ultrarapid metabolizers.^21^^,^^34^ Across different human populations, the normal and intermediate (IM) phenotypes are most common, with normal metabolizers comprising 43–67% of populations and IMs comprising an additional 10–44%.^56^ Here, our focus was primarily on the LoF allele *CYP2D6∗4*. Given the existence of additional non-functional alleles, we anticipate a broader applicability of the findings from this study.

To enable future clinical translation, we screened in total 525 anticancer agents that are either in clinical use or undergoing clinical trials and discovered 12 compounds which show a greater toxicity to cells deficient in CYP2D6 activity, namely AZD-3463 (ALK/IGF1R inhibitor), CYC-116 and PHA-680632 (aurora kinase inhibitor), etoposide (topoisomerase II inhibitor), everolimus and GDC-0349 (mTOR kinase inhibitor), lenvatinib, tyrphostin 9 and WZ-3146 (GFRs inhibitor), talazoparib (PARP enzymes inhibitor), MK-8776 (checkpoint kinase1 inhibitor) and VX-702 (p38 MAP kinase inhibitor), in an engineered HEK293T cell model naturally devoid of any other CYP activity. Additional investigations utilizing *in vitro* models of hepatocellular carcinoma confirmed that talazoparib and MK-8776 consistently displayed cytotoxic effects against cells with compromised CYP2D6 activity. Talazoparib is a clinically used poly (ADP-ribose) polymerase (PARP) inhibitor,^57^ and 64.7% of talazoparib is excreted in the urine with 54.6% as unchanged drug (FDA, reference ID 485951). Talazoparib has also been reported to undergo slow metabolism by the liver,^58^ aligning with our discovery that cancer cells with lower CYP2D6 activity exhibit heightened susceptibility to talazoparib treatment. Since HCCO with lower CYP2D6 activity displayed higher sensitivity to talazoparib, future *in vivo* experiments to validate this finding are warranted.

Moreover, both the MAPK pathway and the PI3K-Akt pathway exhibited significant upregulation in HepG2 CYP2D6 knockout cells, and several growth factor/receptors (PDGFRB, NGFR, TGFA, FGFR1 and FGF21) were identified as co-shared upregulated genes by these two pathways. This finding suggests that the altered activity of CYP2D6 may have broader effects beyond drug metabolism, influencing cell signalling.

The genetic variability that leads to resistance to drug treatment is a key challenge in cancer therapy. For instance, mutations can heighten the activity of crucial signalling pathways, promote cell growth and proliferation while dampening apoptosis, thus contributing to drug resistance.^59^ This includes the upregulation of DNA repair proteins, which may explain why HepG2 HCC cells exhibit relative resistance to talazoparib. However, the CYP2D6-dependent response of HCCO to talazoparib (IC_50_ intermediate 2.17 μM vs normal 7.66 μM), suggests that this therapeutic concept is relevant also at drug concentrations achievable during patient treatment. Moreover, combining talazoparib with currently utilized drugs for liver cancer may potentially enhance treatment outcomes for patients who have lost CYP2D6 activity in their tumor. As this study concerned anticancer drugs that are either used in clinical practice or undergoing clinical trials across various types of cancer, not specifically targeting HCC, future screens tailored specifically toward HCC may result in additional candidate drugs with enhanced efficacy for HCC tumours with CYP2D6 deficiency. This is particularly relevant given the low 3-year overall survival rate, which remains <50%.

We also acknowledge the limitations of this study, including: (i) we only focused on the *CYP2D6∗4* allele, the most common *CYP2D6* LoF alleles. However, considering the existence of various other non-functional *CYP2D6* alleles (∗3, ∗5–∗8, ∗11, ∗12, ∗14–∗16, ∗18–∗21, ∗38, ∗40–∗42, ∗44, ∗51, ∗56, ∗57, ∗60, ∗62, ∗68, ∗69, ∗92, ∗96, ∗99–∗101, ∗114), and that ∼25% of the population has intermediate CYP2D6 activity, we expect that strategies targeting CYP2D6 activity will have widespread applicability; (ii) we encountered challenges in identifying patient-derived organoids having the poor CYP2D6 activity phenotype. We hypothesize that talazoparib or MK-8776 will demonstrate better efficacy in tumors with a poor CYP2D6 phenotype.

Collectively, we have (i) validated earlier findings of deleterious variants present in the human genome; (ii) discovered a set of genes having validated prevalent inactivating alleles in the population; (iii) identified CYP2D6 as a putative target for LOH therapy; and (iv) demonstrated increased cytotoxicity of talazoparib in hepatocellular cancer cell models and organoids. Considering that the incidence of liver cancer worldwide is ∼900,000 cases (IARC 2020), predicted to increase >55% by 2040,^60^ ∼25% of the population (median 10–44%^56^) has the CYP2D6 intermediate phenotype, and the LOH frequency at 22q is 28%, about 50,000 individuals yearly could potentially benefit from LOH therapies targeting lack of CYP2D6 function by 2040. Furthermore, the pursuit of therapies targeting CYP2D6 LoF could hold relevance in the realm of brain tumors. When taking into account the prevalence of glioblastoma in the US and Europe (2.5 per 10,000 individuals, as reported by the brain tumour foundation of Canada), and considering the frequency of chromosome 22q loss in glioblastomas (∼20%), we estimate that >600 individuals each year may have benefit from a therapeutic approach based on loss of heterozygosity. Since talazoparib has been approved by the FDA, it is justified to further explore its potential dependence on CYP2D6 activity in future clinical trials.

## Contributors

Xiaonan Zhang: literature search, study design, data collection, data analysis, data interpretation, data curation, investigation, methodology, software, supervision, validation, visualisation, figures, writing—original draft, and writing; Natallia Rameika: data collection, data analysis, data interpretation, data curation, validation, visualisation and writing; Lei Zhong: data collection, data analysis, data interpretation, investigation, methodology, validation and visualization; Verónica Rendo: data collection, data analysis, methodology, investigation, visualisation and writing; Snehangshu Kundu: data collection, data analysis, methodology, investigation, visualisation and writing; Sandro Nuciforo: data collection, data analysis, methodology, investigation, visualisation, and writing; Jordan Dupuis: data collection, data analysis, methodology, investigation, visualisation, and writing; Muhammad Al Azhar: data collection and data analysis; Ioanna Tsiara: data collection, data analysis and writing; Pauline Seeburger: data collection and data analysis; Shahed Al Nassralla: data analysis and writing; Viktor Ljungström: data analysis and writing; Richard Svensson: data collection and data analysis; Margus Veanes: data analysis and writing; Ivaylo Stoimenov: methodology, data analysis and writing; Per Artursson: investigation, methodology, resources and supervision; Markus H. Heim: investigation, methodology, resources and supervision; Daniel Globisch: investigation, methodology, resources, supervision and writing; Tobias Sjöblom: conceptualisation, literature search, study design, data interpretation, data curation, funding acquisition, methodology, project administration, resources, software, supervision, visualisation, figures, writing – original draft, and writing. All authors have read and approved the final version of the manuscript.

## Data sharing statement

The data supporting the findings of this study are available from the corresponding author upon reasonable request.

## Declaration of interests

The authors declare that they have no competing financial interests or personal relationships that could have influenced the work reported in this paper. The corresponding author of this manuscript, certify that the contributors’ and conflicts of interest statements included in this paper are correct and have been approved by all co-authors.

## Appendix A Supplementary data

Supplementary data 1

Supplementary Figure S1 **a.** Confirmation of HEK293T permanently overexpressing CYP2D6∗1 and CYP2D6∗4 cell model. Expression levels of Flag tagged functional wild-type CYP2D6 and non-functional mutant CYP2D6 were checked by immunoblotting analysis using actin as loading control. **b.** Confirmation of HEK293T cells permanently overexpressing CYP2D6∗1 and CYP2D6∗4, adenovirus overexpressing POR and CYB5A were checked by immunoblotting analysis using actin as loading control. **c.** Detection of CYP2D6 catalytic activity in HEK293T cells stalely overexpressing wild-type (*CYP2D6∗1*) or LoF *CYP2D6* alleles, and transiently overexpressing POR and CYB5A. The formation of dextrorphan was measured at indicated time points after incubation with 10μM CYP2D6 specific substrate dextromethorphan by LC-MS/MS. **d.** Cell growth rate was measured in HEK293T parental, CYP2D6^∗1+POR+B5A^ and CYP2D6^∗4+POR+B5A^ cells. No significantly different growth rates were observed. **e.** The table describes the names, chemical structures, and mechanisms of action of the 12 compounds identified in the screening.

Supplementary Figure S2 **a.** LC/MS/MS chromatogram of dextromethorphan in HepG2 parental and *CYP2D6* KO cell pellets after 90mins incubation with 10μM CYP2D6 specific substrate dextromethorphan. Larger area of dextromethorphan peaks in *CYP2D6* KO cells suggesting that substrate dextromethorphan accumulated in those cells. **b.** String protein–protein interaction networks in drug metabolism-CYP450 pathway. **c.** String protein–protein interaction networks in MAPK signalling pathway. **d.** String protein–protein interaction networks in PI3K-Akt signalling pathway. **e**. The shared genes by MAPK signalling pathway and PI3K-Akt signalling pathway. **f.** IC_50_ of 12 identified hit compounds in the HepG2 parental and *CYP2D6* KO clones. One representative experiment with three technical replicates is shown (mean ± SD). The half-maximal inhibitory concentration (IC_50_) was calculated using Dose-Response inhibition (inhibition vs normalized response) analysis in GraphPad prism. **g**. Dose response to 12 identified hit compounds in the HepG2 parental and *CYP2D6* KO clones. Name of 12 compounds: AZD-3463, CYC-116, etoposide, everolimus, GDC-0349, lenvatinib, MK-8776, PHA-680632, talazoparib, tyrphostin 9, VX-702 and WZ-3146.

Supplementary Figure S3 **a**. The expression level of CYP2D6 in HepG2 parental cells or cells overexpressing either CYP2D6∗1 or CYP2D6∗4 was determined by qPCR. **b**. The expression level of CYP2D6 in HepG2 parental cells or cells overexpressing either CYP2D6∗1 or CYP2D6∗4 was determined by immunoblots. **c.** Kinetic analysis of CYP2D6 catalytic activity in HepG2 cells overexpressing CYP2D6∗1 or LoF CYP2D6∗4. The formation of dextrorphan was measured by LC-MS/MS at indicated time points after incubation with 10μM dextromethorphan. **d**. The formation of dextrorphan in HepG2 parental and CYP2D6∗4 overexpressing cells after 90mins incubation with 10μM dextromethorphan **e**. Dose-response of talazoparib and MK-8776 in HepG2 cells overexpressing CYP2D6∗1 or LoF CYP2D6∗4. One representative experiment with three technical replicates is shown (mean ± SD, unpaired t-test, two-stage step-up (Benjamini, Krieger, and Yekutieli)). **f.** Detection of CYP3A4 catalytic activity in HepG2 parental or CYP3A4 overexpressing cells with or without 2.5μM of the CYP3A4 specific inhibitor ketoconazole (i). **g**. Dose-response for talazoparib and MK-8776 in HepG2 CYP3A4 overexpressing cells absent or present 2.5 μM ketoconazole. **h** and **j.** Detection of CYP1A2 and CYP2C9 catalytic activity in HepG2 parental or overexpressing cells. CYP1A2 inhibitor α-naphthoflavone (5 μM), CYP2C9 inhibitor sulfaphenazole (5 μM). **i** and **k.** Dose-response for talazoparib and MK-8776 in HepG2 CYP1A2 and CYP2C9 overexpressing cells absent or present inhibitors. One representative experiment with three technical replicates is shown (mean ± SD, ∗ P<0.05, ∗∗ P<0.01, ∗∗∗ P<0.001, ∗∗∗∗ P<0.0001, unpaired t-test, Two-stage step-up (Benjamini, Krieger, and Yekutieli).

Supplementary Figure S4 **a.** Representative images for spheroids formed by HEK293T parental and HepG2 parental cells. Bar scale = 300 μm.
