# Supplementary material for: Loss of heterozygosity of CYP2D6 enhances the sensitivity of hepatocellular carcinomas to talazoparib
Source: eBioMedicine. 2024 Oct 4;109:105368. doi: 10.1016/j.ebiom.2024.105368 (PMC11490764; doi:10.1016/j.ebiom.2024.105368)
Supplement: Supplementary Table S1 and S2 [file mmc1.pdf]

Supplementary Table 1. Loss-of-function variants in sensory receptors and keratin-associated proteins.

Gene	Rs accession number	AF	Loss-of-function predicted mechanism
KRT24	rs11309872	0.46	Insertion/deletion causing a shift in the reading frame of a transcript
KRT31	rs117612447	0.01	SNV disrupting a splice site
KRT77	rs201216072	0.02	Insertion/deletion causing a shift in the reading frame of a transcript
KRTAP1-1	rs3213755	0.2	SNV introducing a premature stop codon
	rs200387980	0.05	Insertion/deletion causing a shift in the reading frame of a transcript
KRTAP1-5	rs200596096	0.03	Insertion/deletion causing a shift in the reading frame of a transcript
KRTAP6-2	rs113674499	0.01	Insertion/deletion causing a shift in the reading frame of a transcript
KRTAP8-1	rs200613884	0.05	SNV introducing a premature stop codon
KRTAP10-2	rs113677795	0.02	SNV disrupting a splice site
KRTAP10-4	rs12626886	0.13	SNV disrupting a splice site
	rs75473696	0.01	SNV disrupting a splice site
KRTAP10-6	rs233303	0.22	SNV introducing a premature stop codon
KRTAP13-2	rs877346	0.31	SNV introducing a premature stop codon
OPRM1	rs677830	0.17	SNV introducing a premature stop codon
	rs17174638	0.03	SNV introducing a premature stop codon
	rs34427887	0.03	SNV introducing a premature stop codon
	rs115854517	0.01	SNV disrupting a splice site
OR10AD1	rs58864350	0.21	Insertion/deletion causing a shift in the reading frame of a transcript
OR10H3	rs57132049	0.01	SNV disrupting a splice site
OR10J1	rs12409540	0.08	SNV introducing a premature stop codon
OR10V1	rs499037	0.05	SNV introducing a premature stop codon
OR10X1	rs863362	0.53	SNV introducing a premature stop codon

OR11G2	rs55781225	0.61	Insertion/deletion causing a shift in the reading frame of a transcript
OR13D1	rs113720443	0.01	Insertion/deletion causing a shift in the reading frame of a transcript
OR14C36	rs201185608	0.01	Insertion/deletion causing a shift in the reading frame of a transcript
OR1B1	rs1476860	0.33	SNV introducing a premature stop codon
OR1D5	rs142753580	0.05	Insertion/deletion causing a shift in the reading frame of a transcript
OR1E2	rs200707991	0.02	Insertion/deletion causing a shift in the reading frame of a transcript
	rs11867612	0.22	SNV disrupting a splice site
OR2D3	rs59264610	0.06	Insertion/deletion causing a shift in the reading frame of a transcript
OR2L8	rs10888281	0.76	SNV introducing a premature stop codon
OR2T11	rs149866013	0.02	Insertion/deletion causing a shift in the reading frame of a transcript
OR2T27	rs141113411	0.04	Insertion/deletion causing a shift in the reading frame of a transcript
	rs200669807	0.02	Insertion/deletion causing a shift in the reading frame of a transcript
OR2T4	rs141576206	0.02	Insertion/deletion causing a shift in the reading frame of a transcript
	rs34079073	0.4	Insertion/deletion causing a shift in the reading frame of a transcript
	rs143760725	0.38	Insertion/deletion causing a shift in the reading frame of a transcript
	rs61248663	0.25	Insertion/deletion causing a shift in the reading frame of a transcript
	rs34672924	0.23	Insertion/deletion causing a shift in the reading frame of a transcript
	rs35301588	0.21	Insertion/deletion causing a shift in the reading frame of a transcript
	rs58006779	0.02	Insertion/deletion causing a shift in the reading frame of a transcript
	NT4567235chr11	0.01	Insertion/deletion causing a shift in the reading frame of a transcript

OR2V2	rs140598308	0.07	Insertion/deletion causing a shift in the reading frame of a transcript
OR4C11	rs75423534	0.07	SNV introducing a premature stop codon
OR4C16	rs1459101	0.28	SNV introducing a premature stop codon
OR4C3	rs72473368	0.5	SNV introducing a premature stop codon
OR4D1	rs138082759	0.01	Insertion/deletion causing a shift in the reading frame of a transcript
OR4D10	rs75898556	0.08	SNV introducing a premature stop codon
OR4D6	rs77048571	0.04	Insertion/deletion causing a shift in the reading frame of a transcript
OR4L1	rs112192573	0.5	Insertion/deletion causing a shift in the reading frame of a transcript
OR4M2	rs78256866	0.07	SNV introducing a premature stop codon
OR4P4	rs76160133	0.19	SNV introducing a premature stop codon
OR4X1	rs10838851	0.66	SNV introducing a premature stop codon
OR4X2	rs7120775	0.17	SNV introducing a premature stop codon
OR51B6	rs200982821	0.07	Insertion/deletion causing a shift in the reading frame of a transcript
	rs201581003	0.07	Insertion/deletion causing a shift in the reading frame of a transcript
OR51I1	rs16930998	0.11	SNV introducing a premature stop codon
OR51Q1	rs2647574	0.44	SNV introducing a premature stop codon
OR51V1	rs150098602	0.03	Insertion/deletion causing a shift in the reading frame of a transcript
OR52A1	rs112098990	0.16	Insertion/deletion causing a shift in the reading frame of a transcript
OR52B4	rs11310407	0.33	Insertion/deletion causing a shift in the reading frame of a transcript
OR52I2	rs139794951	0.01	Insertion/deletion causing a shift in the reading frame of a transcript
OR52J3	rs57026471	0.11	SNV introducing a premature stop codon
OR52K2	rs143345847	0.01	Insertion/deletion causing a shift in the reading frame of a transcript
OR52M1	rs145064459	0.01	Insertion/deletion causing a shift in the reading frame of a transcript

	rs200369516	0.01	Insertion/deletion causing a shift in the reading frame of a transcript
	rs201851070	0.01	Insertion/deletion causing a shift in the reading frame of a transcript
OR52N1	rs142442713	0.02	Insertion/deletion causing a shift in the reading frame of a transcript
	rs147003252	0.02	Insertion/deletion causing a shift in the reading frame of a transcript
OR52N4	rs4910844	0.21	SNV introducing a premature stop codon
OR5AC2	rs11369970	0.03	Insertion/deletion causing a shift in the reading frame of a transcript
	rs113439845	0.01	Insertion/deletion causing a shift in the reading frame of a transcript
OR5AR1	rs11228710	0.62	SNV introducing a premature stop codon
OR5AU1	rs74037722	0.01	SNV disrupting a splice site
OR5D16	rs147515254	0.01	Insertion/deletion causing a shift in the reading frame of a transcript
OR5H15	rs35671483	0.03	Insertion/deletion causing a shift in the reading frame of a transcript
OR5K2	rs55639376	0.13	SNV introducing a premature stop codon
OR5L2	rs144106069	0.01	Insertion/deletion causing a shift in the reading frame of a transcript
	rs144335894	0.01	Insertion/deletion causing a shift in the reading frame of a transcript
OR5M1	rs72003051	0.15	Insertion/deletion causing a shift in the reading frame of a transcript
OR5M11	rs17547284	0.04	SNV introducing a premature stop codon
OR6C4	rs59693527	0.05	Insertion/deletion causing a shift in the reading frame of a transcript
	rs138372442	0.04	Insertion/deletion causing a shift in the reading frame of a transcript
	rs142397376	0.04	Insertion/deletion causing a shift in the reading frame of a transcript
	rs148438199	0.04	Insertion/deletion causing a shift in the reading frame of a transcript
	rs75266995	0.04	Insertion/deletion causing a shift in the reading frame of a transcript

OR6C74	rs4522268	0.23	SNV introducing a premature stop codon
OR6Q1	rs34846253	0.13	Insertion/deletion causing a shift in the reading frame of a transcript
OR8B3	rs201661436	0.02	Insertion/deletion causing a shift in the reading frame of a transcript
TAS2R46	rs2708381	0.22	SNV introducing a premature stop codon

Supplementary Table 2. Loss-of-function variants reported in the Online Mendelian Inheritance in Man (OMIM) catalog. OMIM analysis was performed on the initial set of 1,398 LoF variants. The accession numbers for all available phenotypes have been provided.

Gene	Rs accession number	AF	Loss-of-function predicted mechanism	Exon	Alleles	Phenotype*
A2M	rs3832852	0.14	Insertion/deletion disrupting a splice site	18	CTATGG/C	Alzheimer disease (104300).
ADAMTS13	rs121908476	0.01	SNV introducing a premature stop codon	12	C/T	Schulman-Upshaw syndrome (274150).
AMPD1	rs17602729	0.05	SNV introducing a premature stop codon	2	G/A	Myopathy due to myoadenylate deaminase deficiency (615511).
CASP12	rs497116	0.96	SNV introducing a premature stop codon	3	G/A	Susceptibility to sepsis.
CD36	rs3211938	0.03	SNV introducing a premature stop codon	8	T/G	Platelet glycoprotein IV deficiency (608404).<br>Protection against metabolic syndrome (605552).
CLEC7A	rs16910526	0.03	SNV introducing a premature stop codon	6	A/C	Susceptibility to aspergillosis.<br>Candidiasis familial 4 (613108).

CYP2C19	rs4986893	0.01	SNV introducing a premature stop codon	4	G/A	Poor metabolism of proguanil (609535).
CYP2D6	rs3892097	0.11	SNV disrupting a splice site	4	C/T	Poor metabolism of debrisoquine (608902), allelic variant CYP2D6*4 or CYP2D6(B).
	rs5030655	0.01	Insertion/deletion causing a shift in the reading frame of a transcript	3	CA/C	Poor metabolism of debrisoquine (608902), allelic variant CYP2D6*6 or CYP2D6(T).
	rs35742686	0.01	Insertion/deletion causing a shift in the reading frame of a transcript	5	CT/C	Poor metabolism of debrisoquine (608902), allelic variant CYP2D6*3 or CYP2D6(A).
DSC2	rs200056085	0.01	Insertion/deletion causing a shift in the reading frame of a transcript	16	T/TTC	Association with arrhythmogenic right ventricular dysplasia, familial, 11 (610476).
DYX1C1	rs57809907	0.16	SNV introducing a premature stop codon	9	C/A	Susceptibility to dyslexia (127700).
FCN3	rs28357092	0.02	Insertion/deletion causing a shift in the reading frame of a transcript	5	AG/A	Immunodeficiency due to ficolin 3 deficiency (613860).
FLG	rs146466242	0.01	SNV introducing a premature stop codon	2	T/A	Association with psoarisis (603935)/ ichthyosis vulgaris (146700), but contribution to the phenotype has not been confirmed.
	rs61816761	0.01	SNV introducing a premature stop codon	3	G/A	Ichthyosis vulgaris (146700).
FUT2	rs601338	0.32	SNV introducing a premature stop codon	1	G/A	Resistance to Norwalk virus infection.<br>Vitamin B12 plasma level

						quantitative trait locus 1 (612542).
GRIN3B	rs10666583	0.17	Insertion/deletion causing a shift in the reading frame of a transcript	3	G/GCGTT	Association with amyotrophic lateral sclerosis 1 (105400).
LPL	rs328	0.1	SNV introducing a premature stop codon	9	C/G	Lipoprotein lipase polymorphism, associated to elevated LPL or lower HDL levels.
MSR1	rs41341748	0.01	SNV introducing a premature stop codon	5	G/A	Barrett esophagus and/or esophageal adenocarcinoma (614266).
NLRP12	rs104895564	0.01	SNV introducing a premature stop codon	3	G/A	Familial cold autoinflammatory syndrome 2 (611762).
NOD2	rs2066847	0.01	Insertion/deletion causing a shift in the reading frame of a transcript	11	G/GC	Susceptibility to inflammatory bowel disease 1 (266600).
OAS1	rs10774671	0.64	SNV disrupting a splice site	6	G/A	Diabetes mellitus type 1 (222100).<br>Susceptibility to West Nile virus infection (610379).
SLCO1B1	rs71581941	0.01	SNV introducing a premature stop codon	12	C/T	Rotor type hyperbilirubinemia (237450).
TLR5	rs5744168	0.04	SNV introducing a premature stop codon	1	G/A	Susceptibility to Legionnaire disease (608556).<br>Resistance to Systemic Lupus Erythematosus (152700).<br>Resistance to melioidosis (615557).
