# Supplementary material for: Loss of heterozygosity of CYP2D6 enhances the sensitivity of hepatocellular carcinomas to talazoparib
Source: eBioMedicine. 2024 Oct 4;109:105368. doi: 10.1016/j.ebiom.2024.105368 (PMC11490764; doi:10.1016/j.ebiom.2024.105368)
Supplement: Supplementary Table S5 [file mmc4.docx]

**Supplementary Table 5. Genes with prevalent loss-of-function variants were validated using genomAD data resource**.

* The fraction of tumors with LOH at the target locus was estimated from Mertens *et al*., 1997 for the following forms of cancer: breast, colon, kidney, lung, ovarian, head and neck, malignant melanoma, neuroblastoma and neuroglial tumors (1).

|  | **Gene and Chromosomal Location** | **Cancer types with LOH in ≥ 15% of cases*** | **LoF variant and percentage of heterozygotes** | **Effect of LoF variant** |
| --- | --- | --- | --- | --- |
| **1** | ***A2M*** 12p13.31 | Lung: ~20%, Ovarian: ~17% and Melanoma: ~15% | rs1273074647 | splice site disruption |
| **2** | ***AKAP3*** 12p13.32 | Lung: ~20%, Ovarian: ~17% and Melanoma: ~15% | rs2072357 | splice site disruption |
|  |  |  | rs2041291 | frameshift |
|  |  |  | rs200178782 | frameshift |
|  |  |  | rs1990313 | splice site disruption |
|  |  |  | rs12366671 | splice site disruption |
|  |  |  | rs2072355 | splice site disruption |
| **3** | ***ARMC2*** 10p12.2 | Lung: ~24%, Ovarian: ~23%, Melanoma: ~20% and Neuroglial: ~31% | rs10490924 | splice site disruption |
| **4** | ***ATG2B*** 14q32.2 | Lung: ~22% and Ovarian: ~20% | rs3759601 | splice site disruption |
| **5** | ***C12orf60*** 12p12.3 | Ovarian: ~15% | rs11276  rs4236  rs2241221 | splice site disruption |
| **6** | ***C14orf105***  14q22.3 | Kidney: ~22%, Lung: ~21% and Ovarian: ~21% | rs1152522 | splice site disruption |
| **7** | ***C14orf159*** 14q32.11 | Lung: ~22% and Ovarian: ~20% | rs4900072 | splice site disruption |
| **8** | ***C17orf77*** 17q25.1 | Lung: ~17% and Ovarian: ~24% | rs783239  rs58253413 | splice site disruption |
|  |  |  | rs545652 | premature stop codons |
| **9** | ***CCDC182*** 17q22 | Lung: ~17% and Ovarian: ~21% | rs12449409 | splice site disruption |
| **10** | ***CD200R1L*** 3q13.2 | Kidney: ~16% and Melanoma: ~28% | rs771519885 | frameshift |
|  |  |  | rs4682119 | splice site disruption |
| **11** | ***CES5A*** 16q12.2 | Lung: ~17%, Ovarian: ~15% and Melanoma: ~21% | rs11860946 | splice site disruption |
|  |  |  | rs11076126 | splice site disruption |
|  |  |  | rs72810507 | splice site disruption |
| **12** | ***CLDN5*** 22q11.21 | Lung: ~27% | rs885985 | premature stop codons |
| **13** | ***CYP2D6*** 22q13.2 | Lung: ~28%, Ovarian: ~25%, Neuroblastoma: ~18% and Neuroglial: ~22% | rs1058172 | splice site disruption |
|  |  |  | rs3892097 | splice site disruption |
|  |  |  | rs28371704 | splice site disruption |
|  |  |  | rs28371703 | splice site disruption |
| **14** | ***CYP4B1***  **Cytochrome** 11p33 | Breast: ~19%, Colon: ~18%, Lung: ~26%, Ovarian: ~19%, Melanoma: ~18% and Neuroblastoma: ~50% | rs2297809 | splice site disruption |
|  |  |  | rs4646487 | splice site disruption |
|  |  |  | rs3215983 | frameshift |
|  |  |  | rs2297810 | splice site disruption |
|  |  |  | rs4646491 | splice site disruption |
| **15** | ***EBLN2*** 3p13 | Kidney: ~48%, Lung: ~40%, Ovarian: ~18%, Head/neck: ~19% and Melanoma: ~28% | rs2231924 | splice site disruption |
|  |  |  | rs3832186 | frameshift |
|  |  |  | rs2231926 | splice site disruption |
|  |  |  | rs1060584 | splice site disruption |
| **16** | ***EFCAB13*** 17q21.32 | Lung: ~17% and Ovarian: ~21% | rs71377306 | premature stop codons |
|  |  |  | rs4968318 | splice site disruption |
|  |  |  | rs72825679 | splice site disruption |
| **17** | ***EIF3CL*** 16p11.2 | Lung: ~20% and Ovarian: ~18% | rs201261076 | premature stop codons |
| **18** | ***EMR1***  19p13.3 | Lung: ~22% and Ovarian: ~15% | rs330877 | splice site disruption |
|  |  |  | rs330880 | splice site disruption |
|  |  |  | rs897738 | splice site disruption |
|  |  |  | rs443658 | splice site disruption |
|  |  |  | rs370094 | splice site disruption |
|  |  |  | rs466876 | splice site disruption |
|  |  |  | rs457857 | splice site disruption |
|  |  |  | rs373533 | splice site disruption |
|  |  |  | rs461645 | splice site disruption |
|  |  |  | rs7256147 | splice site disruption |
|  |  |  | rs2228539 | splice site disruption |
| **19** | ***ENDOV***  17q25.3 | Lung: ~17% and Ovarian: ~21% | rs34933300 | splice site disruption |
| **20** | ***FUT2*** 19q13.33 | Lung: ~25% and Ovarian: ~18% | rs601338 | premature stop codons |
|  |  |  | rs602662 | splice site disruption |
| **21** | ***GSDMB*** 17q12 | Lung: ~18% and Ovarian: ~21% | rs11078928 | splice site disruption |
| **22** | ***GSTT2*** 22q11.23 | Lung: ~27% | rs5996646 | splice site disruption |
|  |  |  | rs2301423 | splice site disruption |
|  |  |  | rs201176441 | premature stop codons |
| **23** | ***GSTT2B*** 22q11.23 | Lung: ~27% | rs200376763 | premature stop codons |
|  |  |  | rs1622002 | splice site disruption |
| **24** | ***HLA-DQB1*** 6p21.32 | Lung: ~17% and Ovarian: ~15% | rs1130432, rs1140343, rs1140342, rs1049163, rs1063323, rs2647032,  rs1063322, rs1049107, rs1049100,  rs1130399, rs1130398, rs701564,  rs1140320, rs1140319, rs1140318,  rs17412833, rs9274379, rs9274380,  rs9274380, rs1140317, rs1140316,  rs1130392, rs9274384, rs1130387,  rs1130390, rs1130386, rs1071637,  rs1071637, rs1130380, rs1130380,  rs1140313, rs9274395, rs9274397,  rs9274398, rs1049083, rs1063318,  rs750855857, rs281874782,  rs767838657, rs281862065,  rs281862065, rs9274405,  rs1049066, rs1049066,  rs12722115, rs12722115,  rs1130368, rs1130375, rs9274407 | splice site disruption |
|  |  |  | rs1130385 | premature stop codons |
| **25** | ***HTR3D*** 3q27.1 | Ovarian: ~18% and Melanoma: ~28% | rs36092077 | splice site disruption |
|  |  |  | rs73183412 | splice site disruption |
|  |  |  | splice site disruption | splice site disruption |
|  |  |  | rs1000952 | splice site disruption |
| **26** | ***MPP2*** 17q21.31 | Lung: ~17% and Ovarian: ~21% | rs231518 | splice site disruption |
| **27** | ***MPRIP*** 17p11.2 | Colon: ~29%, Lung: ~29% and Ovarian: ~23% | rs749793483 | splice site disruption |
|  |  |  | rs3744137  rs3744124 | splice site disruption |
| **28** | ***MROH2B***  5p13.1 | Lung: ~16% and Ovarian: ~15% | rs2271704, rs10054110, rs16870720,  rs865093, rs13173930, rs325864 | splice site disruption |
|  |  |  | rs1023840 | premature stop codons |
| **29** | ***MUC22*** 6p21.33 | Lung: ~17% and Ovarian: ~15% | rs3094672rs2523898rs4713420  rs12179536rs115709409rs62399429  rs62399430rs62399431rs28360986  rs28360987rs6928386rs111413526  rs62399443rs62401664rs62401665  rs12526820rs12665700rs35464561  rs11756038rs11756039rs9262549  rs12110785  rs10947121 | splice site disruption |
|  |  |  | rs527255810 | premature stop codons |
| **30** | ***NEK3*** 13q14.3 | Lung: ~29% and Ovarian: ~20% | rs3837575 | frameshift |
| **31** | ***NPHP4***  1p36.31 | Breast: ~20%, Colon: ~18%, Lung: ~26%, Ovarian: ~19%, Melanoma: ~18% and Neuroblastoma: ~50% | rs1287637 | splice site disruption |
| **32** | ***OAS1*** 12q24.13 | Melanoma: ~16% | rs1131454 | splice site disruption |
| **33** | ***OBSCN*** 1q42.13 | Lung: ~20% and Ovarian: ~20% | rs1757153, rs1150912, rs1188732,  rs61825301, rs11810627, rs4653942, rs1188729, rs3795801, rs373610,  rs453140, rs369909, rs3795809, rs1188710, rs437129, rs1188697  rs56021350, rs118872, rs1188722, rs435776, rs1188724, rs493945 | splice site disruption |
| **34** | ***PRAMEF2*** 1p36.21 | Breast: ~19%, Colon: ~18%, Lung: ~26%, Ovarian: ~19%, Melanoma: ~18% and Neuroblastoma: ~50% | rs9661554, rs3204790, rs9659529, rs45443899, rs367740116, rs80027487, rs142476002, rs139382628, rs17038667, rs3204798, rs72472698, rs3204805, rs72472699, rs12139546, rs12139550 | splice site disruption |
|  |  |  | rs78738981  rs75411676 | premature stop codons |
| **35** | ***PRB4*** 12p13.2 | Lung: ~20%, Ovarian: ~17% and Melanoma: ~15% | rs1052808  rs59189129  rs12303607  rs1230838 | splice site disruption |
|  |  |  | rs12829245 | premature stop codons |
|  |  |  | rs143553408 | frameshift |
| **36** | ***RAI1*** 17p11.2 | Colon: ~29%, Lung: ~29% and Ovarian: ~23% | rs11649804  rs11649804 | splice site disruption |
|  |  |  | rs749836416 | splice site disruption |
|  |  |  | rs34083643  rs149882795 | frameshift |
| **37** | ***SLC22A14*** 3p22.2 | Kidney: ~49%, Lung: ~44%, Ovarian: ~18%, Head/neck: ~19%, Melanoma: ~29% and Neuroblastoma: ~15% | rs73064822  rs34043027  rs2073714  rs818818  rs818817 | splice site disruption |
| **38** | ***SLC3A1*** 2p21 | Ovarian: ~15% | rs1461067691 | frameshift |
| **39** | ***SLFN12L*** 17q12 | Lung: ~18% and Ovarian: ~21% | rs4796089  rs3744372  rs2304968  rs2304967 | splice site disruption |
| **40** | ***SPATA6L*** 9p24.1 | Lung: ~38%, Ovarian: ~18%, Melanoma: ~18% and Neuroglial: ~19% | rs10974657 | splice site disruption |
| **41** | ***SPERT***  13q14.13 | Lung: ~29% and Ovarian: ~20% | rs80072371 | premature stop codons |
| **42** | ***TBC1D31*** 8q24.13 | Lung: ~21% and Ovarian: ~21% | rs10101626 | splice site disruption |
| **43** | ***TNK1*** 17p13.1 | Colon: ~31%, Lung: ~35%, Ovarian: ~24% and Melanoma: ~15% | rs3744549  rs6503018 | splice site disruption |
| **44** | ***TMPRSS3*** 21q22.3 | Lung: ~26% and Ovarian: ~24% | rs2839500  rs12270001 | splice site disruption |
|  |  |  | rs2276122  rs34966432 | splice site disruption |
| **45** | ***TOR1AIP1*** 1q25.2 | Lung: ~16% | rs609521  rs17279712 | splice site disruption |
|  |  |  | rs2245425 | splice site disruption |
| **46** | ***UBE2NL***  Xq27.3 | Lung: ~26%, Ovarian: ~30%, Neuroblastoma: ~24% and Neuroglial: ~20% | rs237520 | premature stop codons |
| **47** | ***ZNF419*** 19q13.43 | Lung: ~25% and Ovarian: ~18% | rs2074076  rs2074077 | splice site disruption |
|  |  |  | rs2074071 | splice site disruption |
| **48** | ***ZNF681*** 19p12 | Lung: ~22% and Ovarian: ~15% | rs1852432  rs7245561 | splice site disruption |
|  |  |  | rs61397759 | frameshift |
| **49** | ***ZNF812***  19p13.2 | Lung: ~24% and Ovarian: ~19% | rs113307466 | splice site disruption |
|  |  |  | rs762006678 | frameshift |
| **50** | ***ZNF860***  3p23 | Kidney: ~49%, Lung: ~44%, Ovarian: ~18%, Melanoma: ~29% and Neuroblastoma: ~15% | rs13064905  rs13087612  rs13065048 | splice site disruption |
|  |  |  | rs4639011 | premature stop codons |
| **51** | ***ZNF880*** 19q13.41 | Lung: ~25% and Ovarian: ~18% | rs324125  rs8104808  rs8104812  rs14048 | splice site disruption |
|  |  |  | rs34678014 | splice site disruption |
|  |  |  | rs34470614 | frameshift |

**Analysis procdures:**


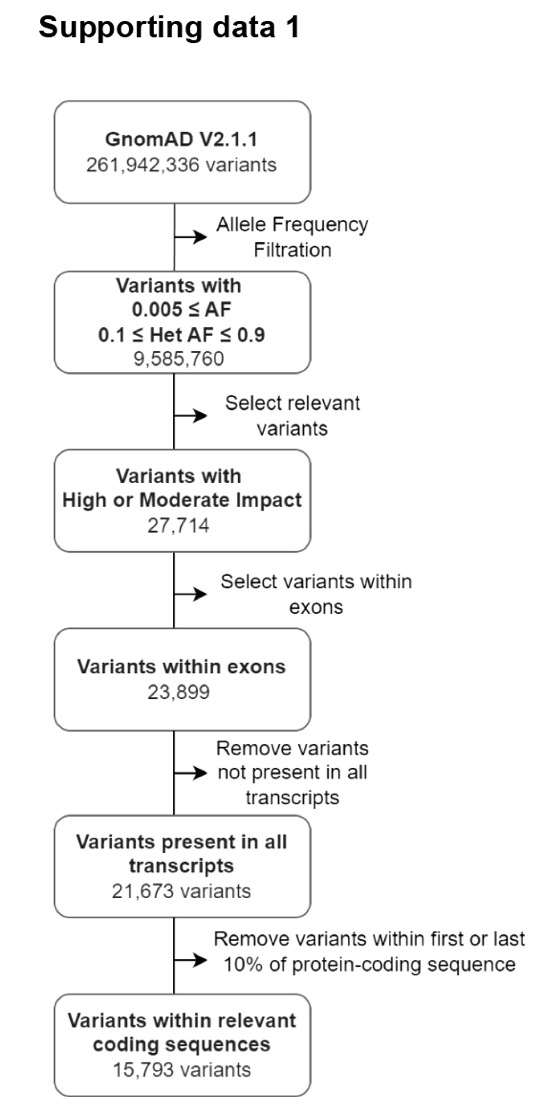


To further validate the initial findings which were based on 1000 Genomes phase 1 data (Release 3, 2012-04-30), genomAD was utilized as an additional resource for human genetic variation data (2). Data was downloaded from the Broad Institute in the form of gnomAD v2.1.1, containing 261,942,336 total variants across 125,748 exomes and 15,708 whole genomes, all mapped to the GRCh37/hg19 reference sequence (3). Annotation of the data was achieved via the SnpEff software package. Impact annotations provided by SnpEff were used to initially reduce the volume of variants, retaining only variants annotated as having ‘high’ or ‘moderate’ impact (4). To enable desired filtration of the data, the consensus coding DNA sequence definitions of human protein coding exons were retrieved via NCBI RefSeq (Update, 2024-02-12) accessed through UCSCs genome browser (5). The exon definitions were used to select all SNVs and indels in protein-coding regions and in splice junctions. As before the selection of splice site variants was restricted to mutations spanning the canonical dinucleotides belonging to splice donor or acceptor sites. These variants were then filtered to remove those with allele frequency less than 0.5% and were additionally constrained to have heterozygosity between 10-90%. Variants outside splice sites occurring within the first or last 10% of the protein coding sequence were discarded alongside any which were not present in all gene transcripts.

Flowchart of LOH target validation

**Reference:**

1. Mertens, F., Johansson, B., Hoglund, M. & Mitelman, F. Chromosomal imbalance maps of malignant solid tumors: a cytogenetic survey of 3185 neoplasms. *Cancer Res* **57**, 2765-2780 (1997).
2. Nonchev Kalin, gnomAD_DB, (2023), GitHub repository, https://github.com/KalinNonchev/gnomAD_DB.git
3. Karczewski, K.J., Francioli, L.C., Tiao, G. et al. The mutational constraint spectrum quantified from variation in 141,456 humans. Nature 581, 434–443 (2020). https://doi.org/10.1038/s41586-020-2308-7
4. "A program for annotating and predicting the effects of single nucleotide polymorphisms, SnpEff: SNPs in the genome of Drosophila melanogaster strain w1118; iso-2; iso-3.", Cingolani P, Platts A, Wang le L, Coon M, Nguyen T, Wang L, Land SJ, Lu X, Ruden DM. Fly (Austin). 2012 Apr-Jun;6(2):80-92. PMID: 22728672
5. Pruitt KD, Tatusova T, Maglott DR. NCBI Reference Sequence (RefSeq): a curated non-redundant sequence database of genomes, transcripts and proteins. Nucleic Acids Res. 2005 Jan 1;33(Database issue):D501-4. doi: 10.1093/nar/gki025. PMID: 15608248; PMCID: PMC539979.
