# Supplementary material for: Loss of heterozygosity of CYP2D6 enhances the sensitivity of hepatocellular carcinomas to talazoparib
Source: eBioMedicine. 2024 Oct 4;109:105368. doi: 10.1016/j.ebiom.2024.105368 (PMC11490764; doi:10.1016/j.ebiom.2024.105368)
Supplement: Supplementary Figure S1 — a. Confirmation of HEK293T permanently overexpressing CYP2D6∗1 and CYP2D6∗4 cell model. Expression levels of Flag tagged functional wild-type CYP2D6 and non-functional mutant CYP2D6 were checked by immunoblotting analysis using actin as loading control. b. Confirmation of HEK293T cells permanently overexpressing CYP2D6∗1 and CYP2D6∗4, adenovirus overexpressing POR and CYB5A were checked by immunoblotting analysis using actin as loading control. c. Detection of CYP2D6 catalytic activity in HEK293T cells stalely overexpressing wild-type (CYP2D6∗1) or LoF CYP2D6 alleles, and transiently overexpressing POR and CYB5A. The formation of dextrorphan was measured at indicated time points after incubation with 10μM CYP2D6 specific substrate dextromethorphan by LC-MS/MS. d. Cell growth rate was measured in HEK293T parental, CYP2D6∗1+POR+B5A and CYP2D6∗4+POR+B5A cells. No significantly different growth rates were observed. e. The table describes the names, chemical structures, and mechanisms of action of the 12 compounds identified in the screening. [file mmc8.pdf]

Supplementary Figure 1

a

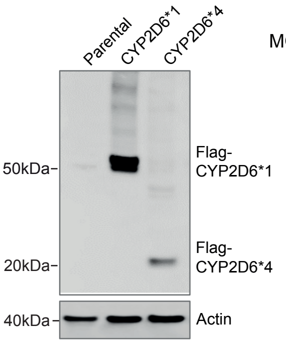

b

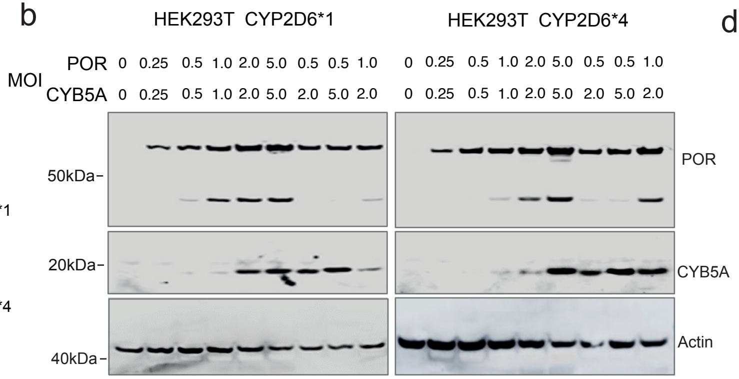

d

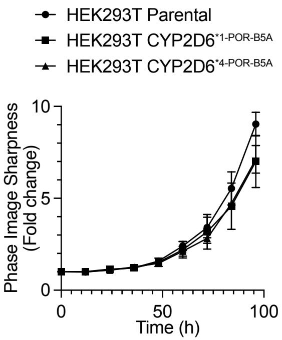

c

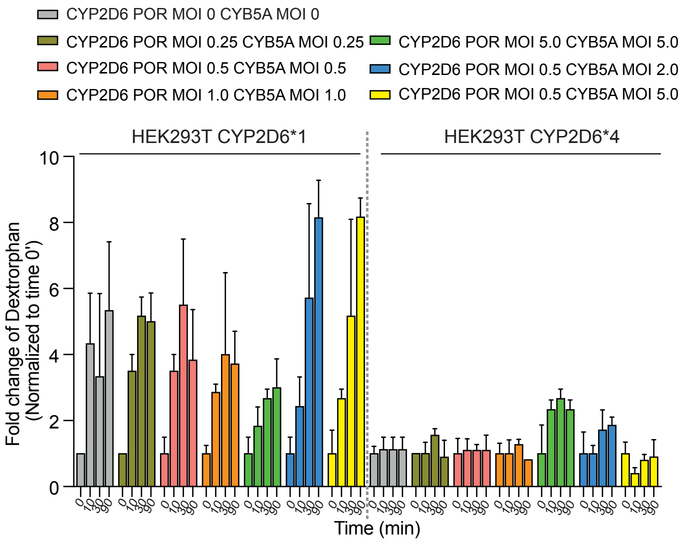

e

Information of 12 identified compounds

	Name	Structure	Mechanism of action		Name	Structure	Mechanism of action
1	AZD-3463	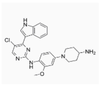	ALK/IGF 1R inhibitor	7	MK-8776	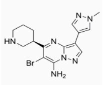	Checkpoint kinase1 (Chk1) inhibitor
2	CYC-116	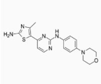	Aurora A and aurora B inhibitor	8	PHA-680632	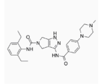	Aurora kinase inhibitor
3	Etoposide	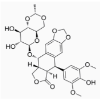	Topoisomerase II inhibitor	9	Talazoparib	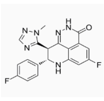	Poly (ADP-ribose) polymerase (PARP) enzymes inhibitor
4	Everolimus	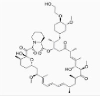	Rapamycin mTOR kinase inhibitor	10	Tyrphostin A9	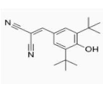	PDGFR inhibitor
5	GDC-0349	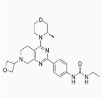	ATP-competitive mTOR inhibitor	11	VX-702	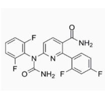	p38 MAP kinase inhibitor
6	Lenvatinib	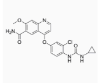	VEGFR, FGFR, PDGFR, KIT, and RET inhibitor	12	WZ-3146	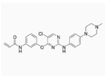	EGFR inhibitor
