# Supplementary material for: Loss of heterozygosity of CYP2D6 enhances the sensitivity of hepatocellular carcinomas to talazoparib
Source: eBioMedicine. 2024 Oct 4;109:105368. doi: 10.1016/j.ebiom.2024.105368 (PMC11490764; doi:10.1016/j.ebiom.2024.105368)

# Supplementary Figure 2

a

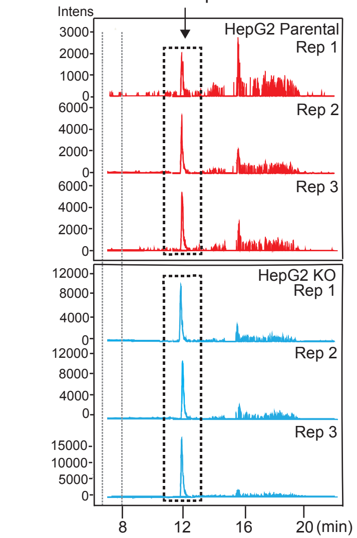


f

$IC_{50}$ of 12 preliminary hit compounds

| $IC_{50}$ (µM) | AZD-3463 | CYC-116 | WZ-3146 | Etoposide | Everolimus | GDC-0349 | Lenvatinib | MK-8776 | PHA-680632 | Talazoparib | Tyrphostin 9 | VX-702 |
|---|---|---|---|---|---|---|---|---|---|---|---|---|
| HepG2 Parental | 0.59 | 7.7 | 3.7 | 6.0 | 20.1 | 33.6 | 63.1 | 13.9 | 38.8 | 64.8 | 4.5 | >50 |
| HepG2 CYP2D6 KO C1 | 0.51 | 5.2 | 3.3 | 5.5 | 12.2 | 16.2 | 50.2 | 3.4 | 22.9 | 24.8 | 3.9 | >50 |
| HepG2 CYP2D6 KO C2 | 0.51 | 6.2 | 2.9 | 5.5 | 15.1 | 17.9 | 52.9 | 2.7 | 20.7 | 16.2 | 2.8 | >50 |

g

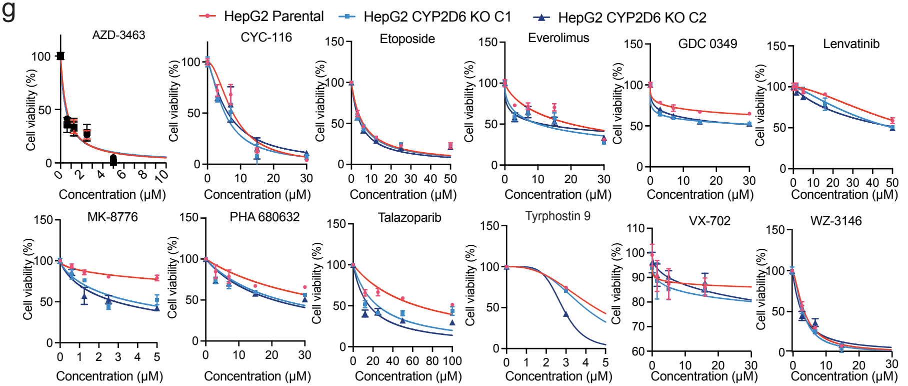


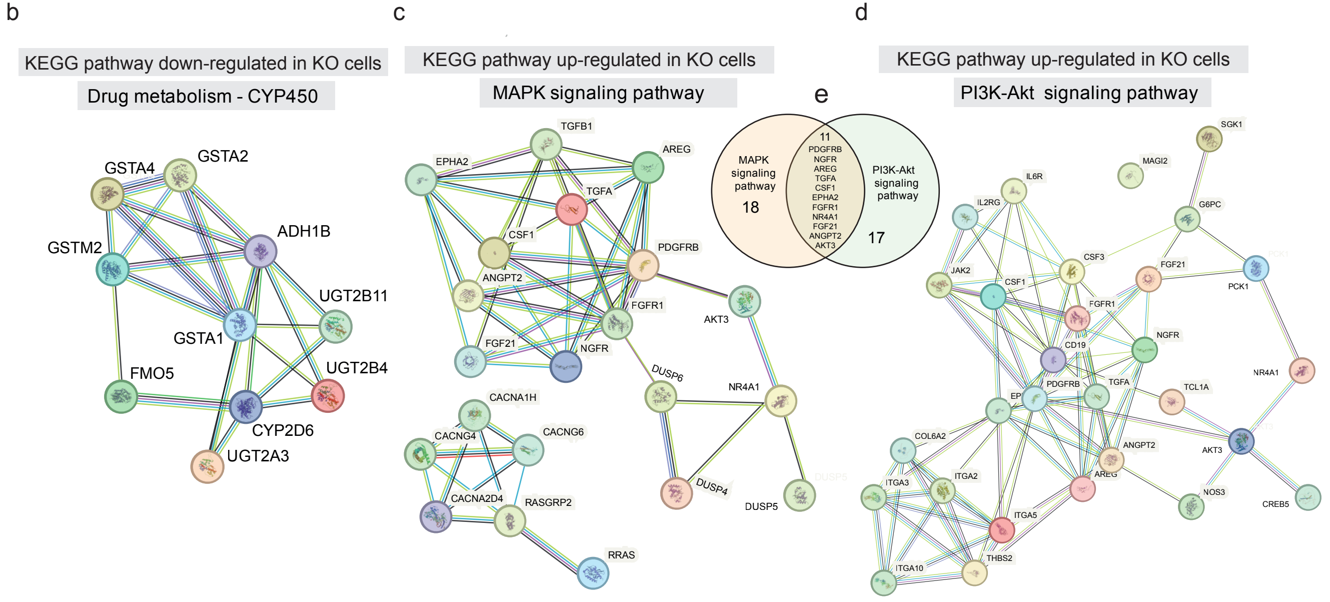

Supplement: Supplementary Figure S2 — a. LC/MS/MS chromatogram of dextromethorphan in HepG2 parental and CYP2D6 KO cell pellets after 90mins incubation with 10μM CYP2D6 specific substrate dextromethorphan. Larger area of dextromethorphan peaks in CYP2D6 KO cells suggesting that substrate dextromethorphan accumulated in those cells. b. String protein–protein interaction networks in drug metabolism-CYP450 pathway. c. String protein–protein interaction networks in MAPK signalling pathway. d. String protein–protein interaction networks in PI3K-Akt signalling pathway. e. The shared genes by MAPK signalling pathway and PI3K-Akt signalling pathway. f. IC50 of 12 identified hit compounds in the HepG2 parental and CYP2D6 KO clones. One representative experiment with three technical replicates is shown (mean ± SD). The half-maximal inhibitory concentration (IC50) was calculated using Dose-Response inhibition (inhibition vs normalized response) analysis in GraphPad prism. g. Dose response to 12 identified hit compounds in the HepG2 parental and CYP2D6 KO clones. Name of 12 compounds: AZD-3463, CYC-116, etoposide, everolimus, GDC-0349, lenvatinib, MK-8776, PHA-680632, talazoparib, tyrphostin 9, VX-702 and WZ-3146. [file mmc9.pdf]
