# Supplementary figures and images for: Loss of heterozygosity of CYP2D6 enhances the sensitivity of hepatocellular carcinomas to talazoparib
Source: eBioMedicine. 2024 Oct 4;109:105368. doi: 10.1016/j.ebiom.2024.105368 (PMC11490764; doi:10.1016/j.ebiom.2024.105368)

# Supplementary Figure 3

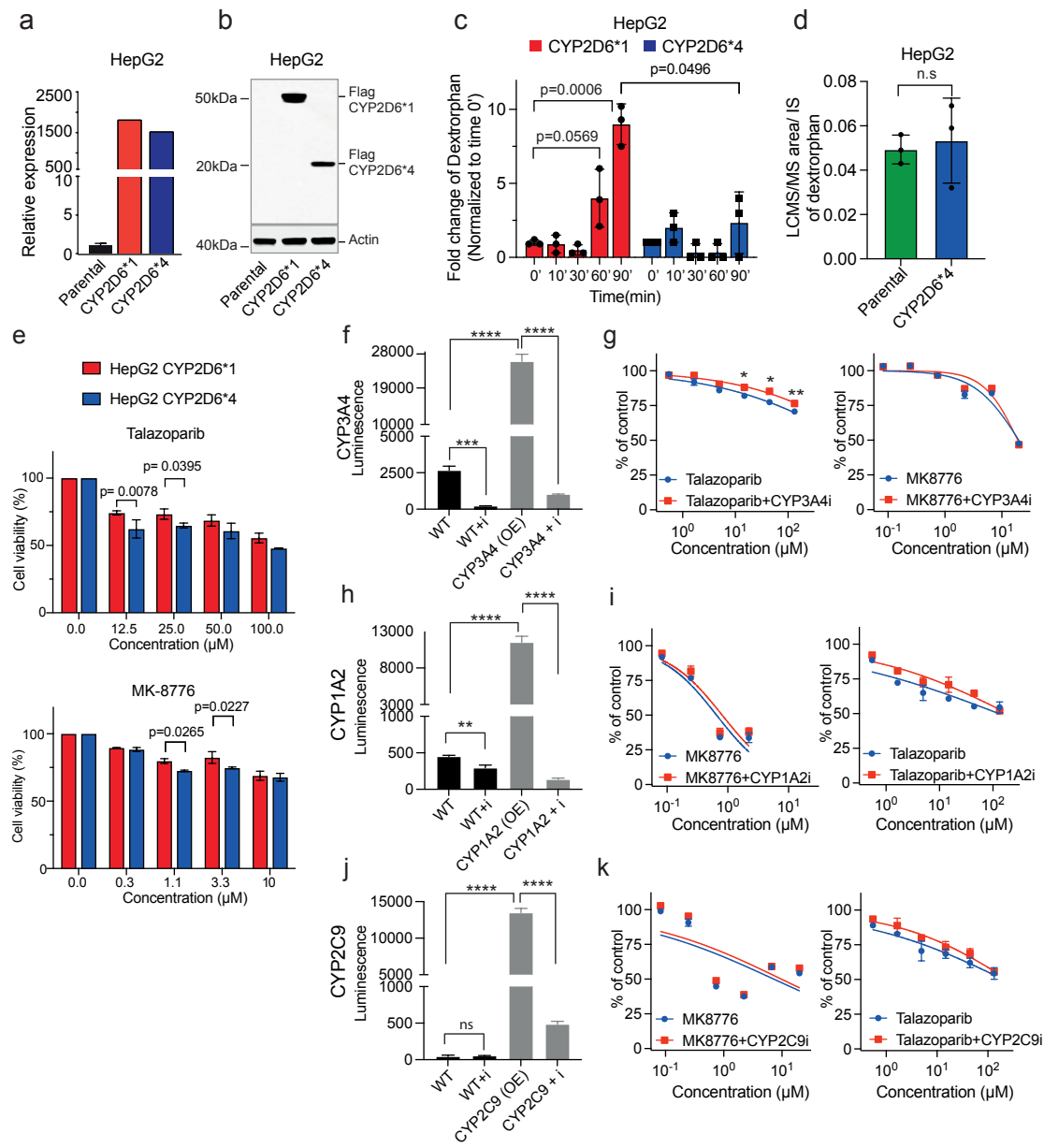

Supplement: Supplementary Figure S3 — a. The expression level of CYP2D6 in HepG2 parental cells or cells overexpressing either CYP2D6∗1 or CYP2D6∗4 was determined by qPCR. b. The expression level of CYP2D6 in HepG2 parental cells or cells overexpressing either CYP2D6∗1 or CYP2D6∗4 was determined by immunoblots. c. Kinetic analysis of CYP2D6 catalytic activity in HepG2 cells overexpressing CYP2D6∗1 or LoF CYP2D6∗4. The formation of dextrorphan was measured by LC-MS/MS at indicated time points after incubation with 10μM dextromethorphan. d. The formation of dextrorphan in HepG2 parental and CYP2D6∗4 overexpressing cells after 90mins incubation with 10μM dextromethorphan e. Dose-response of talazoparib and MK-8776 in HepG2 cells overexpressing CYP2D6∗1 or LoF CYP2D6∗4. One representative experiment with three technical replicates is shown (mean ± SD, unpaired t-test, two-stage step-up (Benjamini, Krieger, and Yekutieli)). f. Detection of CYP3A4 catalytic activity in HepG2 parental or CYP3A4 overexpressing cells with or without 2.5μM of the CYP3A4 specific inhibitor ketoconazole (i). g. Dose-response for talazoparib and MK-8776 in HepG2 CYP3A4 overexpressing cells absent or present 2.5 μM ketoconazole. h and j. Detection of CYP1A2 and CYP2C9 catalytic activity in HepG2 parental or overexpressing cells. CYP1A2 inhibitor α-naphthoflavone (5 μM), CYP2C9 inhibitor sulfaphenazole (5 μM). i and k. Dose-response for talazoparib and MK-8776 in HepG2 CYP1A2 and CYP2C9 overexpressing cells absent or present inhibitors. One representative experiment with three technical replicates is shown (mean ± SD, ∗ P<0.05, ∗∗ P<0.01, ∗∗∗ P<0.001, ∗∗∗∗ P<0.0001, unpaired t-test, Two-stage step-up (Benjamini, Krieger, and Yekutieli). [file mmc10.pdf]

# Supplementary Figure 4

a

HEK293T

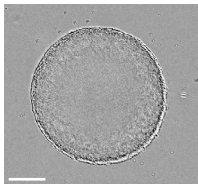

HepG2

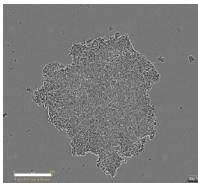

Supplement: Supplementary Figure S4 — a. Representative images for spheroids formed by HEK293T parental and HepG2 parental cells. Bar scale = 300 μm. [file mmc11.pdf]
